# The inflammasome NLRP3 plays a dual role on mouse corpora cavernosa relaxation

**DOI:** 10.1038/s41598-019-52831-0

**Published:** 2019-11-07

**Authors:** Rafael S. Fais, Fernanda L. Rodrigues, Camila A. Pereira, Allan C. Mendes, Fabíola Mestriner, Rita C. Tostes, Fernando S. Carneiro

**Affiliations:** 1 Departments of Pharmacology, Ribeirao Preto Medical School, University of Sao Paulo, Sao Paulo, Brazil; 2 Departments of Physiology, Ribeirao Preto Medical School, University of Sao Paulo, Sao Paulo, Brazil

**Keywords:** Cardiovascular biology, Urology

## Abstract

NLRP3 plays a role in vascular diseases. Corpora cavernosa (CC) is an extension of the vasculature. We hypothesize that NLRP3 plays a deleterious role in CC relaxation. Male C57BL/6 (WT) and NLRP3 deficient (NLRP3^−/−^) mice were used. Intracavernosal pressure (ICP/MAP) measurement was performed. Functional responses were obtained from CC strips of WT and NLRP3^−/−^ mice before and after MCC950 (NLRP3 inhibitor) or LPS + ATP (NLRP3 stimulation). NLRP3, caspase-1, IL-1β, eNOS, nNOS, guanylyl cyclase-β1 (GCβ1) and PKG1 protein expressions were determined. ICP/MAP and sodium nitroprusside (SNP)-induced relaxation in CC were decreased in NLRP3^−/−^ mice. Caspase-1, IL-1β and eNOS activity were increased, but PKG1 was reduced in CC of NLRP3^−/−^. MCC950 decreased non-adrenergic non-cholinergic (NANC), acetylcholine (ACh), and SNP-induced relaxation in WT mice. MCC950 did not alter NLRP3, caspase-1 and IL-1β, but reduced GCβ1 expression. Although LPS + ATP decreased ACh- and SNP-, it increased NANC-induced relaxation in CC from WT, but not from NLRP3^−/−^ mice. LPS + ATP increased NLRP3, caspase-1 and interleukin-1β (IL-1β). Conversely, it reduced eNOS activity and GCβ1 expression. NLRP3 plays a dual role in CC relaxation, with its inhibition leading to impairment of nitric oxide-mediated relaxation, while its activation by LPS + ATP causes decreased CC sensitivity to NO and endothelium-dependent relaxation.

## Introduction

The corpora cavernosa (CC) are the primary structure responsible for penile erection. These structures depend on the abundant blood supply to carry out their function^1–4^. The CC tonus is modulated by the activity of the sympathetic (SNS) and parasympathetic (PNS) autonomic nervous system^4–7^. The SNS is responsible for the maintenance of the flaccid state of the penis through the release of noradrenaline (NA), which leads to the activation of calcium-dependent and -independent signaling pathways that promote CC smooth muscle contraction. On the other hand, the PNS induces CC relaxation, by nitric oxide (NO) release, directly from nitrergic nerve-endings containing neuronal NO synthase (nNOS) or the activation of the endothelial NO synthase (eNOS) isoform by acetylcholine from cholinergic nerve-endings.

The erectile function is closely linked with vascular function, mainly due to the similarity of the structures that form the cavernosal spaces and the arterioles^4–7^.

Several studies suggest that the immune system play a role in CC tone modulation through the release and activation of inflammatory mediators^8–10^. Toll-like receptors (TLR) overactivation impairs the reactivity of CC mainly by the release of proinflammatory cytokines such as tumor necrosis factor (TNF)-α and interleukin (IL)-1β^11,12^. These mediators stimulate CC contractile responses, through increased RhoA/Rho Kinase activity^11^, and reduction of NO bioavailability, which decreases the relaxation of CC^12^. Elevated levels of these cytokines may also lead to CC structural changes in chronic conditions^13–19^.

IL-1β is the product of inflammasome activation^20,21^. The inflammasome is a multiprotein complex of the innate immune system, and the nucleotide-binding oligomerization domain leucine-rich repeat containing pyrin 3 (NLRP3) is the most studied receptor of this complex. NLRP3 depends on two signals for its activation. First, nuclear factor kappa B (NF-κB) activation, mainly via TLR4, releases inactive forms of cytokines as well as components of the inflammasome complex^22^. The second signal occurs through a membrane perturbation, such as pore-forming proteins in the membrane, ATP-P2X channels overactivation, increased reactive oxygen species (ROS) generation, phagolysosomal or mitochondrial destabilization^23^. The second signal leads to NLRP3 oligomerization and assembly of the inflammasome complex, which promotes caspase-1 auto-cleavage and subsequent processing and release of the active forms of IL-1β and IL-18^22–26^. The components of the inflammasome are closely linked with the onset of vascular dysfunction, leading to functional and/or structural damage^27–31^. Based on these data, we hypothesized that NLRP3 plays a detrimental role in the modulation of the CC relaxation, which may predispose to erectile dysfunction (ED) development.

## Materials and Methods

### Animals

Male C57BL/6 (WT) and NLRP3^−/−^ mice were housed in a room with controlled temperature (20 to 22 °C) and on light/dark cycles of 12 hours with free access to standard chow and filtered water. Mice were used at 10 to 12 weeks of age (25 g). All experimental animal protocols followed the regulations of the National Council on Animal Experimental Control (CONCEA, Brazil) and were approved by the Ethics Committee on Animal Experimentation (CEUA n° 005/2015-1) at Ribeirao Preto Medical School.

### Drugs and solutions

Physiological Krebs Henseleit buffer of the following composition was used: NaCl 130 mM, KCl 4.7 mM, KH_2_PO_4_ 1.18 mM, MgSO_4_.7H_2_O 1.17 mM, NaHCO_3_ 14.9 mM, EDTA 0.026 mM, CaCl_2_.2H_2_O 1.6 mM and D-glicose 5.55 mM. The incubations were performed with MCC950 (1 µM^32^ Cayman Chemical 17510; diluted in 5% DMSO and 95% deionized water), lipopolysaccharide (LPS) (1 µg/mL; diluted in deionized water), adenosine 5-triphosphate (ATP) [(2 mM, Sigma-Aldrich A6144; diluted in deionized water).

To evaluate the relaxation, the following drugs were used: acetylcholine (ACh) (100 pM–10 µM; diluted in deionized H_2_O), sodium nitroprusside (SNP) (10 pM–30 µM; NO donor), phenylephrine (10 µM), guanethidine (30 µM), atropine (1 µM) and L-NAME (100 mM) purchased from Sigma Chemical Co. (St. Louis, MO). Stock solutions were prepared in deionized water and stored in aliquots at −20 °C; dilutions were made up immediately before use.

### Cavernosal tissue reactivity

Cavernosal strips were isolated and mounted in 5 mL-myograph chambers (Danish Myo Technology, Aarhus, Denmark) containing Krebs Henseleit buffer continuously bubbled with a mixture of 95% O_2_ and 5% CO_2_ and maintained at 37 °C. The tissues were stretched to a resting force of 2.5 mN and allowed to equilibrate for 60 min Changes in isometric force were recorded using a PowerLab/8SP data acquisition system (Chart software, version 5.2; ADInstruments, Colorado Springs, CO). A solution containing high concentration of potassium chloride (KCl, 120 mM) was added to the organ baths at the end of the equilibration period to verify the contractile ability of the preparations. The CC strips were divided into three groups: group 1 – WT and NLRP3^−/−^ CC strips; group 2 – WT CC strips incubated with NLRP3 MCC950 or vehicle for 2 hours; group 3 – WT and NLRP3^−/−^ CC strips incubated with vehicle or LPS for 4 hours followed by stimulation with ATP for 10 minutes (LPS + ATP).

Relaxation responses were evaluated by cumulative concentration-response curves for ACh and SNP in CC strips contracted with phenylephrine. All SNP concentration-response curves were performed after incubation with L-NAME (100 µM; diluted in deionized H_2_O) to prevent interference of basal NO production. Electrical field stimulation (EFS) (20 V, 0.2 to 32 Hz) was performed to determine non-adrenergic non-cholinergic (NANC)-mediated relaxations. Briefly, it was performed an incubation (30 minutes) with guanethidine (30 µM) and atropine (1 µM); the strips were then contracted with phenylephrine (10 µM). After reaching a plateau, the EFS was performed to observe the relaxation. Each stimulation lasted 10 s, and an interval between stimuli was allowed until full recovery of the resting tension.

### Western blot assay

The CC were isolated, cleaned from surrounding fat tissue, snap frozen in liquid nitrogen and homogenized in a lysis buffer [50 mM Tris/HCl, 150 mM NaCl, 1% Nonidet P40, 1 mM EDTA, 1 μg/ml leupeptin, 1 μg/ml pepstatin, 1 μg/ml aprotinin, 1 mM sodium orthovanadate, 1 mM phenylmethanesulfonyl fluoride (PMSF), and 1 mM sodium fluoride]^33^. Protein concentration was determined by the Bradford assay. Spectra multicolor broad range protein ladder (10 to 260 KDa) was used as a protein standard. Aliquots with 30 µg of proteins were prepared and separated by electrophoresis at 100 V for 2 hours at 4 °C in 10% polyacrylamide gel (SDS-PAGE) and transferred for 1 hour to a nitrocellulose membrane at 100 V at 4 °C. Gels were stained with Coomassie blue and membranes with Ponceau red 2% to demonstrate the transference efficiency. Nonspecific binding sites of the membrane to the primary antibodies were blocked with 5% bovine serum albumin (BSA) solution for 1 hour at room temperature. The primary antibodies described below were incubated for 12 hours at 4 °C, and the secondary antibodies were incubated for 1 hour at room temperature. Protein bands visualization were obtained by chemiluminescence after ECL reaction (Amersham ECL Prime Western Blotting Detection Reagent) and image capture performed on ImageQuant 350 gel imager (GE Healthcare, Piscata Way, NJ, USA). The densitometric quantification was performed by ImageJ® software. Membranes were stripped with restore western blot stripping buffer (Thermo) for 45 minutes at 37 °C. The following antibodies were used in the study: NLRP3 (MAB7578, diluted 1:500, R&D), caspase-1 (IMG-5028, diluted 1:500, Imgenex), IL-1β [(H-153)-SC-7884, diluted 1:500, Santa Cruz Biotechnology], phospho-eNOS [(ser1177)-9571S, diluted 1:500, Cell Signal], eNOS (9572 S, diluted 1:500, Cell Signaling), nNOS (4234 S, diluted 1:1.000, Cell Signaling), PKG1 (3248 S, diluted 1:500, Cell Signaling), guanylyl cyclase α (GCα) (AB50358, diluted 1:1.000, Abcam), guanylyl cyclase β (GCβ) (SAB4501344, diluted 1:1.000, Sigma-Aldrich). The glyceraldehyde-3-phosphate dehydrogenase (GAPDH) (G9545, diluted 1:5.000, Sigma-Aldrich) expression was used as endogenous control for normalization of all proteins. Membranes were then incubated with the following secondary antibodies: goat anti-mouse IgG H&L (AB6789, diluted 1:10.000, Abcam), goat anti-rabbit IgG H&L (AB6721, diluted 1:10.000, Abcam), rabbit anti-rat IgG (AB6703, diluted 1:3.000, Abcam).

### *In vivo* measurements of intracavernosal pressure and mean arterial pressure

The animals were anesthetized with 2% isoflurane in 100% oxygen (2 L/min). Then, the left carotid artery and right CC of each mouse were cannulated for continuous monitoring of mean arterial pressure (MAP) and intracavernous pressure (ICP), respectively. Finally, the cavernosal nerve (CNV) was stimulated electrically with silver electrodes at different frequencies (5 V, 1 ms pulses, and frequencies between 0.2 and 20 Hz) to induce changes in ICP. During the stimulation these animals were maintained anesthetized with isoflurane 1% in 100% oxygen (2 L/min).

### Statistical analysis

The results were analyzed by the Student’s t-test. Values of p less than 0.05 were considered statistically significant. The contractile responses were represented as the force developed from the baseline tonus in millinewtons (mN) normalized by the dry weight (g) of individual CC strips in a given number (n) of experiments. On the other hand, relaxation responses were expressed as the percentage change from pre-contraction induced by phenylephrine. Concentration-effect curves were submitted to non-linear regression analysis using the GraphPad Prism program (GraphPad Prism 6.0; GraphPad Software Inc., San Diego, CA, USA). Agonist potency and maximal response were expressed as pEC_50_ (negative logarithm of molar concentration producing 50% of the maximal response) and E_max_ (maximal effect produced by the agonist), respectively. Statistical analysis of the E_max_ and pEC_50_ values was performed using nonlinear regression followed by Student’s t-test.

## Results

### Effect of NLRP3 deletion on the erectile function of mice

**Figure 1:**
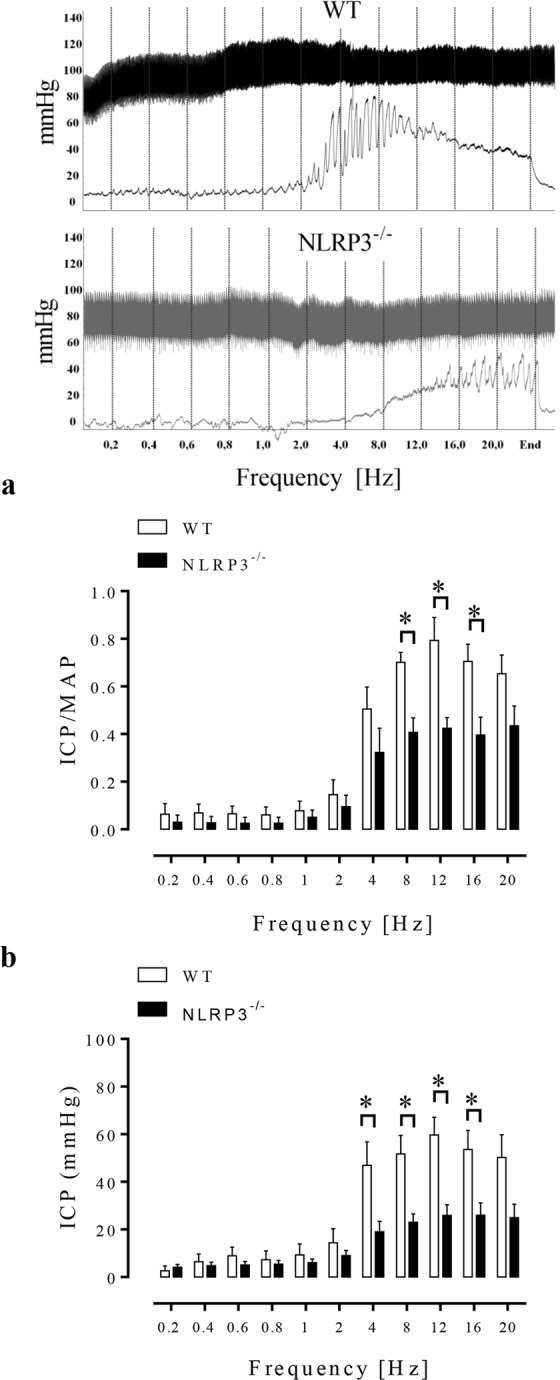
Effect of NLRP3 deletion in the ICP/MAP ratio (**a**) and raw ICP data (**b**). Graph depicts the ICP/MAP ratio and raw ICP response to cavernosal nerve stimulation assessed over a range of frequencies (0.2–20 Hz). Data represent the mean ± SEM values of the groups (graph in the left). Representative tracings showing changes in intracavernosal pressure (bottom traces) and blood pressure (top traces) in response to electrical stimulation of the cavernosal nerve stimulation (right of the figure). *p < 0.05 compared to WT group. n = 5–6. The comparison of each frequency value for the ICP/MAP ratio and raw ICP of WT (white bars) and NLRP3^−/−^ (black bars) was performed by Student’s t-test. ICP = intracavernosal pressure; MAP = mean arterial pressure.

The first set of experiments shows that *in vivo* measurement of ICP demonstrated that electrical stimulation of the cavernosal nerve induced frequency-dependent ICP changes in NLRP3^−/−^ and WT mice. The ICP/MAP ratio at 8, 12 and 16 Hz was decreased in NLRP3^−/−^ mice (Fig. 1a). In addition, the ICP alone at 4, 8, 12 and 16 Hz was decreased in NLRP3^−/−^ mice (Fig. 1b). These data suggest that NLRP3^−/−^ mice display decreased erectile function.

### NLRP3 downstream signaling pathway and CC reactivity in NLRP3 knockout mice

**Figure 2:**
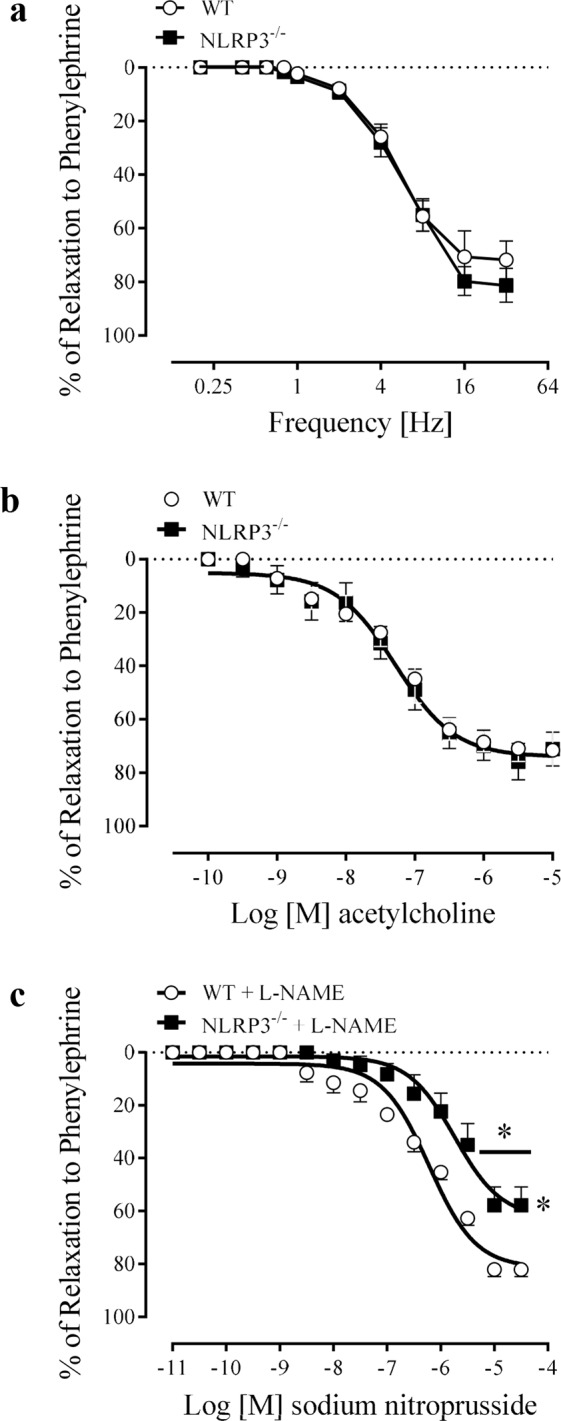
Frequency-response curves for NANC-induced relaxation **(a)**, concentration-effect curves to acetylcholine (100 pM–10 µM) **(b)** and sodium nitroprusside (10 pM–30 µM) **(c)** in CC strips of WT (white spheres) and NLRP3^−/−^ (black squares) mice. Data represent the mean ± SEM values of the groups. *p < 0.05 compared to WT group. n = 4–6. The comparison of each frequency value for NANC-induced relaxation, pEC_50_ and Emax parameters was performed by Student’s t-test.

No differences in NANC- or ACh-induced relaxation (Fig. 2a,b) were observed between CC strips of WT and NLRP3^−/−^ mice. However, SNP-mediated relaxation was decreased in CC strips of NLRP3^−/−^ mice compared to WT (Fig. 2c). The values of pEC_50_ and Emax for the relaxation induced by ACh and SNP are described in Table 1.

**Table 1:** Values of Emax (%) and pEC_50_ for the concentration-effect curves to ACh and SNP in CC from WT and NLRP3^−/−^ mice under conditions of stimulation (LPS + ATP) or inhibition (MCC950) of the NLRP3 inflammasome.

Drug	WT vehicle	WT MCC950		
**Pharmacological inhibition of NLRP3 with MCC950**				
ACh				
pEC_50_	6.90 ± 0.09	6.32 ± 0.09*		
Emax	80.50 ± 8.91	53.93 ± 3.87*		
SNP				
pEC_50_	7.25 ± 0.06	6.67 ± 0.08*		
Emax	100 ± 3.40	100 ± 6.18		
**Concentration-effect curves in CC from NLRP3**^−/−^ **mice**				
**Drug**	**WT**	**NLRP3**^−/−^		
ACh				
pEC_50_	7.27 ± 0.10	7.26 ± 0.11		
Emax	71.61 ± 7.84	75.86 ± 6.84		
SNP				
pEC_50_	6.22 ± 0.06	5.73 ± 0.10*		
Emax	80.02 ± 2.45	63.11 ± 5.23*		
**Activation of NLRP3 with LPS + ATP**				
**Drug**	**WT** **vehicle**	**WT** **LPS + ATP**	**NLRP3**^−/−^ **vehicle**	**NLRP3**^−/−^ **LPS + ATP**
ACh				
pEC_50_	6.74 ± 0.07	6.90 ± 0.09	6.88 ± 0.06	6.90 ± 0.12
Emax	79.28 ± 5.21	54.60 ± 5.69*	68.29 ± 2.10	61.41 ± 10.66
SNP				
pEC_50_	7.29 ± 0.14	6.61 ± 0.06*	7.04 ± 0.09	6.68 ± 0.10
Emax	100 ± 4.80	100 ± 2.64	100 ± 3.25	100 ± 3.99

Values are mean ± SEM (n = 4 to 6 in each group). *p < 0.05 WT vehicle vs WT MCC950, WT vs NLRP3^−/−^ or WT vehicle vs WT LPS + ATP. The comparison of pEC_50_ and Emax parameters was performed by Student’s t-test.

**Figure 3:**
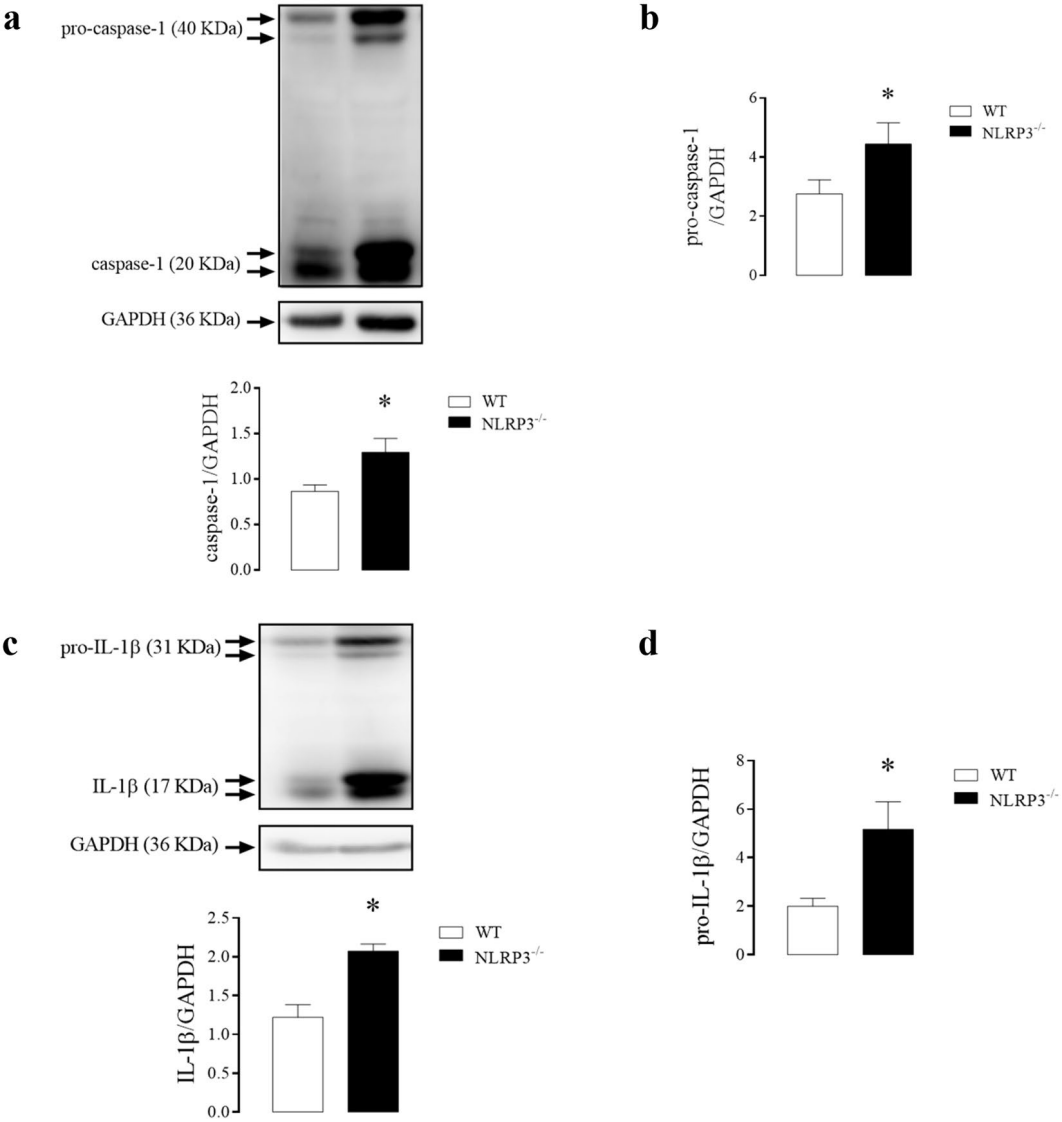
Densitometric analysis of caspase-1 **(a)**, pro-caspase-1 **(b)**, IL-1β **(c)** and pro-IL-1β **(d)** in CC strips of WT (white bar) and NLRP3^−/−^ (black bar) mice. The expression of GAPDH was determined and used as the internal control. The bars represent the mean ± SEM values of protein expression. *p < 0.05 compared to WT group. n = 6–8. The comparison of protein expression was performed by Student’s t-test.

The CC of NLRP3^−/−^ mice displayed increased expression of caspase-1 (Fig. 3a), pro-caspase-1 (Fig. 3b), IL-1β (Fig. 3c) and pro-IL-1β (Fig. 3d) when compared to WT mice.

### Effect of NLRP3 deletion on the signaling pathways of CC relaxation

**Figure 4:**
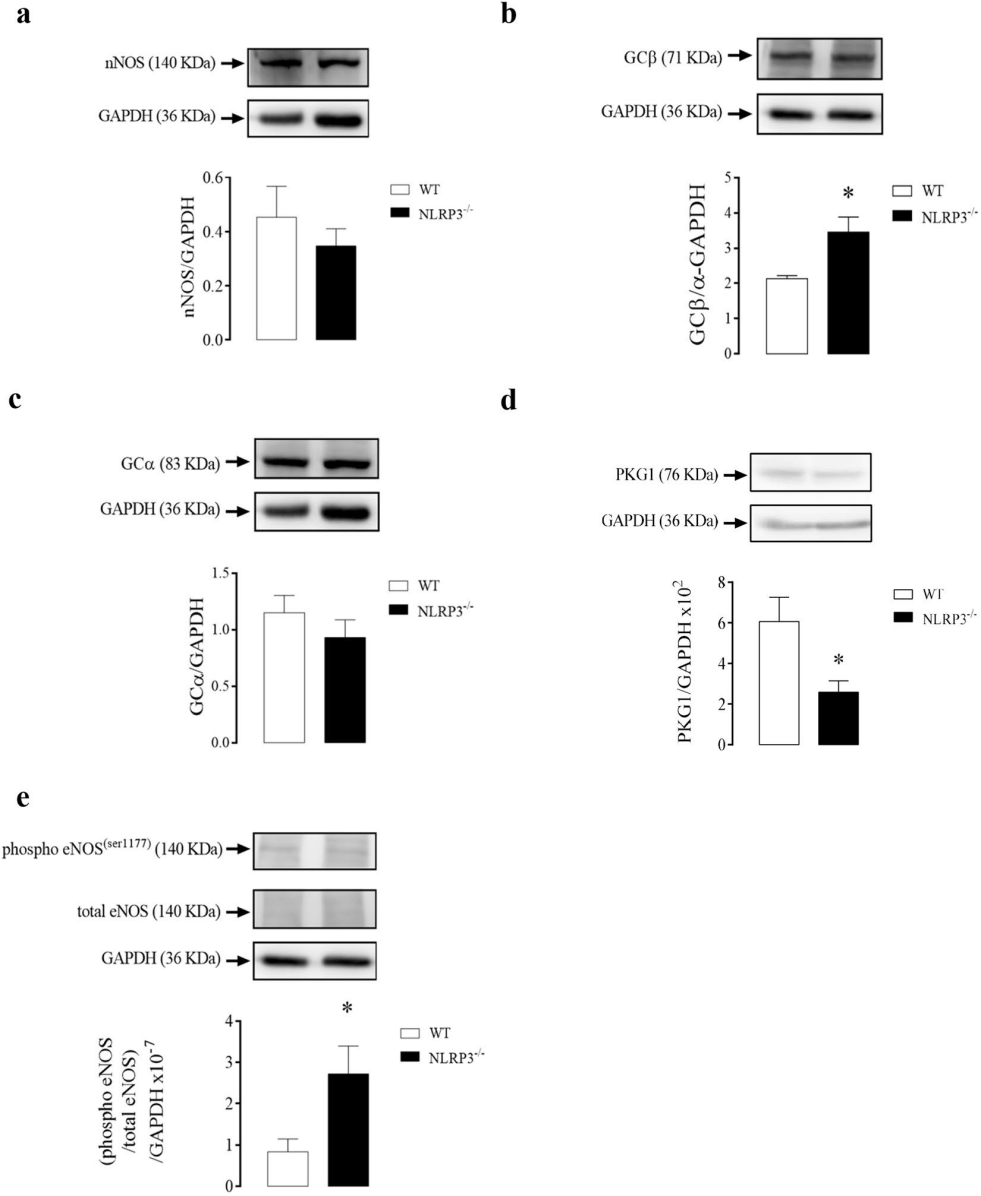
Densitometric analysis of nNOS **(a)**, GCβ **(b)**, GCα **(c)**, PKG1 **(d)** and eNOS phosphorylation **(e)** expressions in CC strips of WT (white bars) and NLRP3^−/−^ (black bars) mice. The expression of GAPDH was determined and used as the internal control. Data represent the mean ± SEM values of protein expression. *p < 0.05 compared to WT group. n = 4–6. The comparison of protein expression was performed by Student’s t-test.

There were no changes in nNOS (Fig. 4a) protein expression in CC of NLRP3^−/−^ mice. On the other hand, GCβ expression (Fig. 4b), but not GCα (Fig. 4c) was increased in CC of NLRP3^−/−^ mice when compared to control animals. Also, CC strips of NLRP3^−/−^ mice showed decreased expression of PKG1 (Fig. 4d) and increased eNOS phosphorylation (Fig. 4e).

### Effect of NLRP3 pharmacological inhibition on CC reactivity

**Figure 5:**
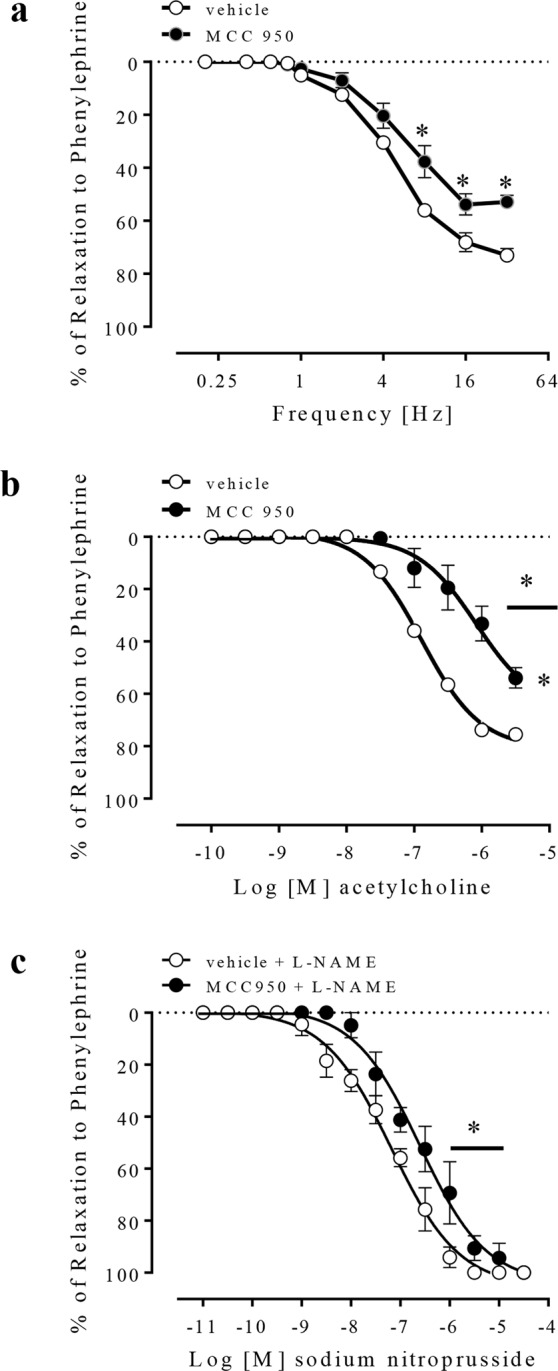
Frequency-response curves for NANC-induced relaxation **(a)**, concentration-effect curves to acetylcholine (100 pM–3 µM) **(b)** and sodium nitroprusside (10 pM–30 µM) **(c)**, in vehicle- (white spheres) or MCC950-treated (1 µM, black spheres) CC strips from WT mice. Data represent the mean ± SEM values of the groups. *p < 0.05 compared to vehicle group. n = 5–6. The comparison of each frequency value for NANC-induced relaxation, pEC_50_ and Emax parameters was performed by Student’s t-test.

NANC-induced relaxation was decreased in CC strips in the presence of a NLRP3 inhibitor (Fig. 5a). Similarly, endothelium-dependent relaxation to ACh (Fig. 5b) and endothelium-independent relaxation to SNP (Fig. 5c) were decreased in CC strips treated with MCC950. The values of pEC_50_ and Emax for the relaxation induced by ACh and SNP are described in Table 1.

**Figure 6:**
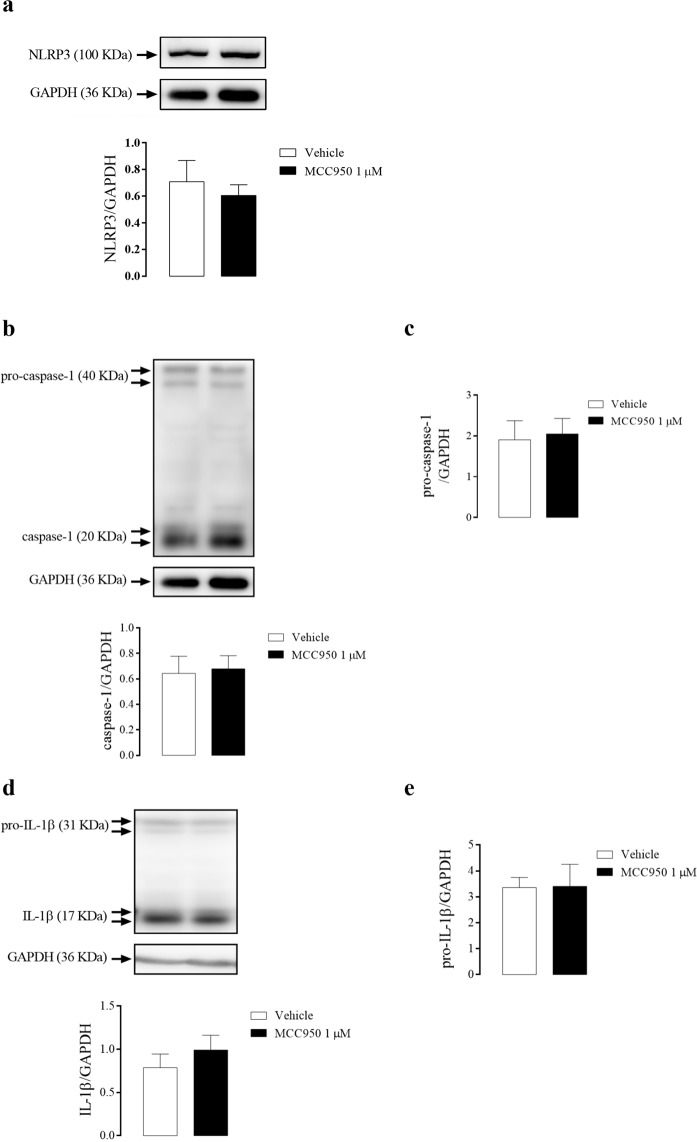
Densitometric analysis of NLRP3 **(a)**, caspase-1 **(b)**, pro-caspase-1 **(c)**, IL-1β **(d)** and pro-IL-1β **(e)** expression in CC strips of WT mice incubated with MCC950 (1 µM, black bars) or vehicle (white bars). The expression of GAPDH was determined and used as the internal control. The bars represent the mean ± SEM values of protein expression. n = 5–6. The comparison of protein expression was performed by Student’s t-test.

Inhibition of NLRP3 with MCC950 did not alter the expression of NLRP3 (Fig. 6a), caspase-1 (Fig. 6b), pro-caspase-1 (Fig. 6c), IL-1β (Fig. 6d), or pro-IL-1β (Fig. 6e) in the CC of WT mice.

### Effect of NLRP3 pharmacological inhibition on the signaling pathways of CC relaxation

**Figure 7:**
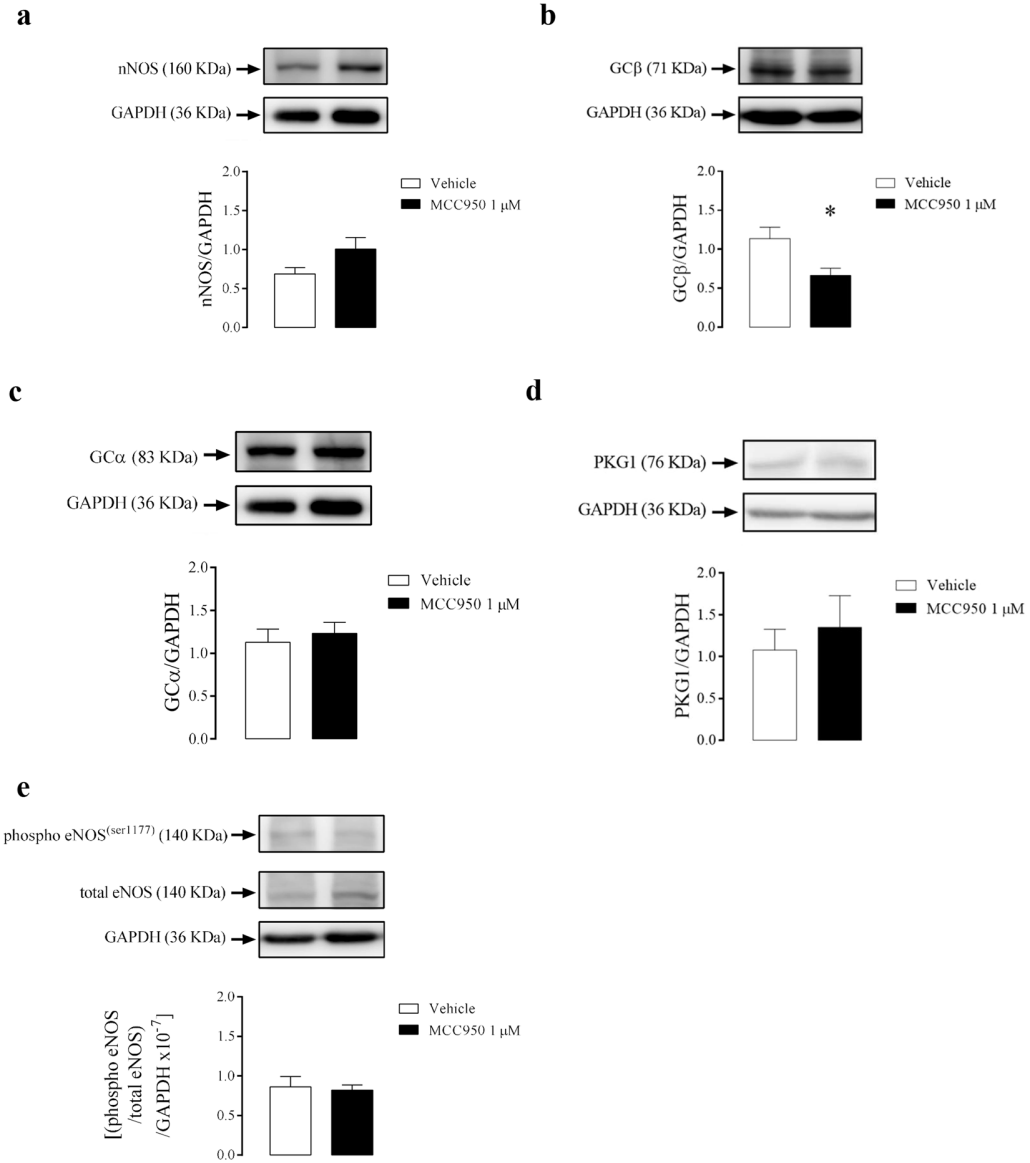
Densitometric analysis of nNOS **(a)**, GCβ **(b)**, GCα **(c)**, PKG1 **(d)** and eNOS phosphorylation **(e)** expressions in CC strips of WT mice incubated with MCC950 (1 µM, black bars) or vehicle (white bars). The expression of GAPDH was determined and used as the internal control. Data represent the mean ± SEM values of protein expression. *p < 0.05 compared to vehicle group. n = 5–6. The comparison of protein expression was performed by Student’s t-test.

There was no change in the protein expression levels of nNOS (Fig. 7a) and reduction of GCβ (Fig. 7b) in CC strips from WT mice after incubation with MCC950. Nevertheless, MCC950 incubation did not alter the expression of GCα (Fig. 7c), PKG1 (Fig. 7d) and eNOS phosphorylation (Fig. 7e) in CC strips from WT mice.

### Effect of NLRP3 activation on CC reactivity

**Figure 8:**
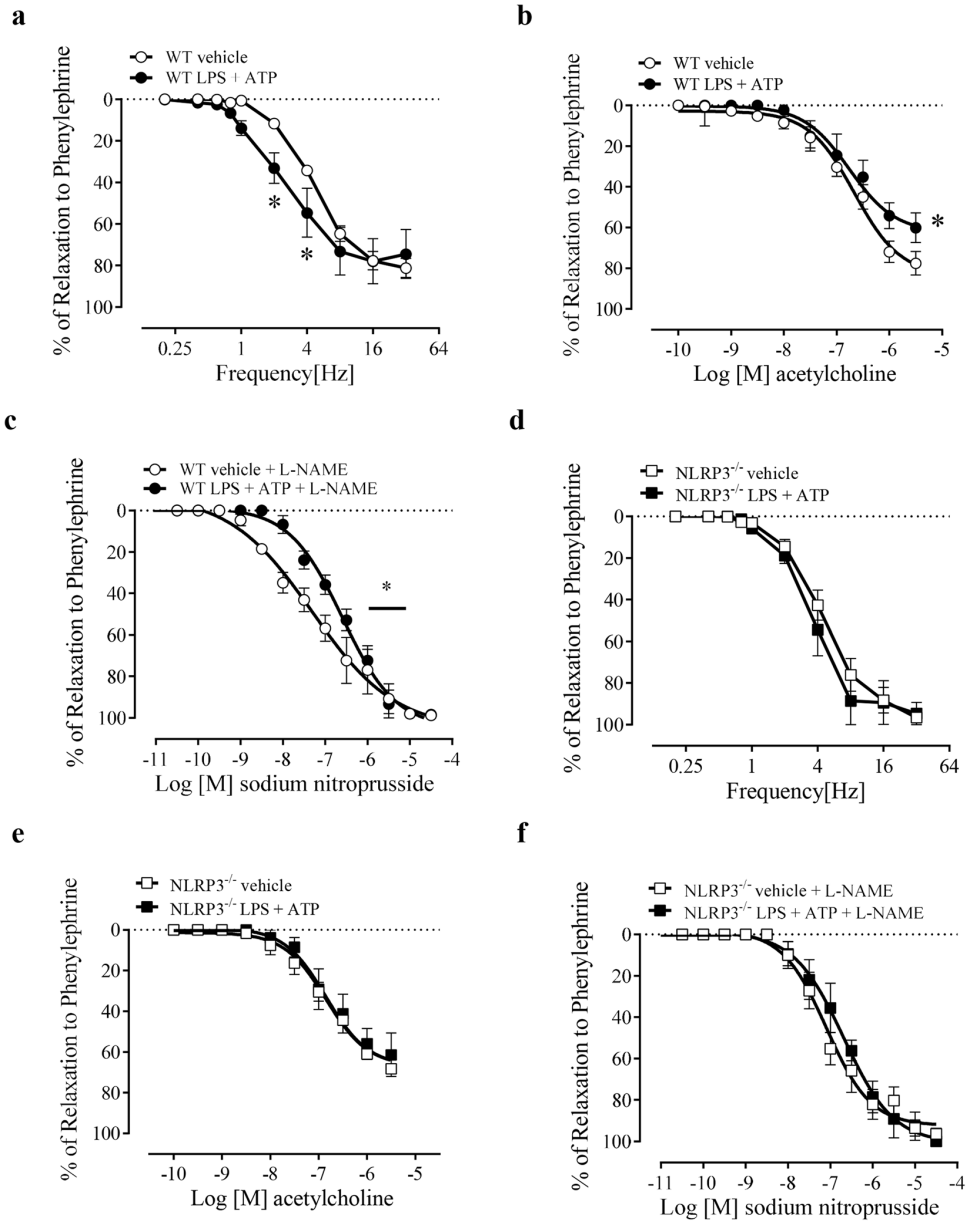
Frequency-response curves for NANC-induced relaxation (**a**,**d**), concentration-effect curves to acetylcholine (100 pM–3 µM) (**b**,**e**) and sodium nitroprusside (10 pM–30 µM) (**c**,**f**) in mice CC strips of WT vehicle (white spheres), WT incubated with LPS + ATP (1 µg/mL + 2 mM) (black spheres), NLRP3^−/−^ vehicle (black square) and NLRP3^−/−^ incubated with LPS + ATP (1 µg/mL + 2 mM) (white square). Data represent the mean ± SEM values of the groups. *p < 0.05 compared to WT LPS + ATP group. n = 5–6. The comparison of each frequency values for NANC-induced relaxation, pEC_50_ and Emax parameters was performed by Student’s t-test.

The activation of NLRP3, with LPS + ATP incubation, increased NANC- potency (Fig. 8a), reduced the ACh- maximal response (Fig. 8b) and SNP-mediated relaxation potency (Fig. 8c) in CC of WT mice. However, these functional changes were prevented in CC from NLRP3^−/−^ mice (Fig. 8d–f). The values of pEC_50_ and Emax induced by ACh and SNP are described in Table 1.

**Figure 9:**
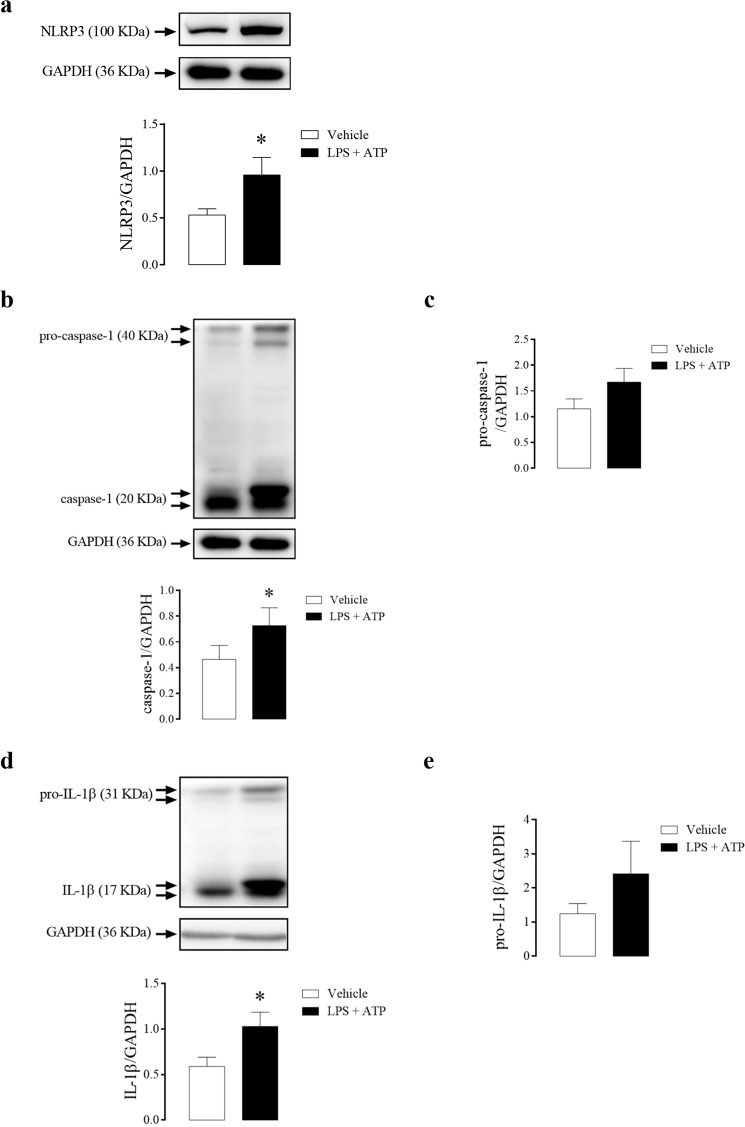
Densitometric analysis of NLRP3 **(a)**, caspase-1 **(b)**, pro-caspase-1 **(c)**, IL-1β **(d)** and pro-IL-1β **(e)** expression in CC strips of WT mice incubated with LPS + ATP (1 µg/mL + 2 mM, black bars) or vehicle (white bars). The expression of GAPDH was determined and used as the internal control. The bars represent the mean ± SEM values of protein expression. *p < 0.05 compared to respective control group. n = 5–6. The comparison of protein expression was performed by Student’s t-test.

The stimulation of mice CC with LPS followed by ATP increased NLRP3 protein expression (Fig. 9a), caspase-1 (Fig. 9b), but not pro-caspase-1 (Fig. 9c) expression, and also increased IL-1β (Fig. 9d) and a tendency to increase pro-IL-1β (Fig. 9e) expression.

### Effect of NLRP3 activation on the signaling pathways of CC relaxation

**Figure 10:**
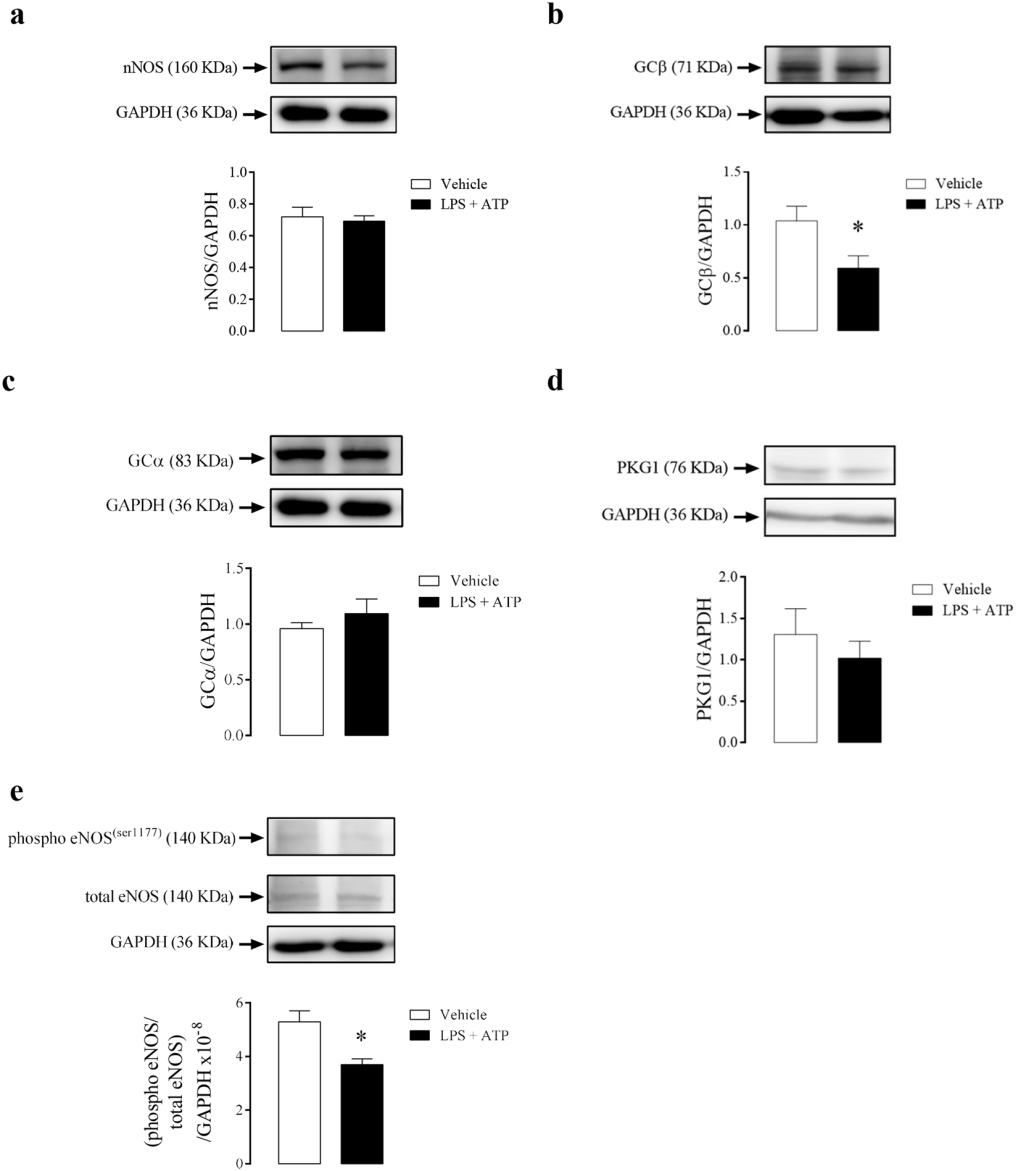
Densitometric analysis of nNOS **(a)**, GCβ **(b)**, GCα **(c)**, PKG1 **(d)** and eNOS phosphorylation **(e)** in CC strips of WT mice incubated with LPS + ATP (1 µg/mL + 2 mM, black bars) or vehicle (white bars). The expression of GAPDH was determined and used as the internal control. Data represent the mean ± SEM values of protein expression. *p < 0.05 compared to vehicle group. n = 5–6. The comparison of protein expression was performed by Student’s t-test.

Activation of NLRP3, by LPS + ATP, did not change nNOS (Fig. 10a) expression. Nevertheless, it reduced GCβ (Fig. 10b) without changes in the GCα (Fig. 10c) and PKG1 (Fig. 10d) protein expression when compared to control animals. Also, the CC strips of WT mice showed and decreased phosphorylation of eNOS (Fig. 10e).

### Supplemental data

All the Western blotting full representative membranes and the GAPDH statistics are present in the supplemental data (Figs s1–s9).

## Discussion

The results of the present study indicate that NLRP3 has a dual role in mice CC relaxation, with its inhibition leading to impairment of NO-mediated relaxation, while its overactivation causes a decreased cavernosal smooth muscle sensitivity to NO and endothelium-dependent relaxation. NLRP3, an essential member of the innate immune system, overactivation or inhibition impairs, respectively, the nitric oxide- and endothelium-mediated CC relaxation. Indeed, NLRP3 plays a crucial role in the cardiovascular system, since vascular cells can detect and respond to damage-associated molecular patterns (DAMPs) or pathogen-associated molecular patterns (PAMPs) via TLRs and NLRs. Therefore, it promotes the release of cytokines, chemokines and dilating hormones^31,34,35^, which facilitates the transfer and migration of leukocytes to the lesion site^36–38^. Erectile dysfunction (ED) and cardiovascular diseases share the same risk factors^39^. As an example, the sustained presence of low-grade inflammatory mediators in patients with ED and coronary artery diseases is well documented^40^. Nevertheless, it was still unknown whether the machinery that produces inflammatory mediators contributes to modulate the tonus of CC.

Clinical and experimental evidence show that increased activity of the innate immune system is implicated in the pathogenesis of ED^11,41^, atherosclerosis^42^, acute coronary syndrome^43^, and cerebrovascular accidents^44^. The exact mechanism by which the innate immune system acts in the genesis of ED or cardiovascular diseases has not yet been fully elucidated. Perhaps the contribution of the immune system to cardiovascular diseases and ED development is the exacerbation of the inflammatory process, which may contribute to the generation of vascular and CC lesions^10,45^. Further support to this idea is the fact that the increased levels of proinflammatory cytokines are closely linked to the genesis of ED^11^.

Considering the facts mentioned above, the present manuscript determined whether NLRP3, a protein involved in IL-1β and IL-18 maturation, contributes to CC relaxation modulation. Initially, it was demonstrated that NLRP3 is not only expressed and displayed in a constitutive manner, but it can also be activated in CC of mice. These findings are determined by the following facts: (1) there are active caspase-1 and IL-1β in mice CC at basal conditions; and (2) LPS + ATP stimulus is able to increased NLRP3 expression, caspase-1 and IL-1β release in CC, which is similar to NLRP3 activation in cells of the immune system^46,47^. The basal activity of NLRP3 in CC suggests that it may modulate CC function at physiological levels. Also, its activation may contribute to functional changes at pathophysiological states. Since NLRP3 is expressed and active in CC, we decided to dig deeper in our research on the effect of NLRP3 inhibition in CC.

The CC from NLRP3^−/−^ mice showed higher expression of caspase-1, pro-caspase-1, pro-IL-1β, and IL-1β. This effect may occur due to overactivation of other NLR or TLR evoked by the absence of NLRP3 in CC. The inflammasome is a dynamic multiprotein complex, whereas different components of the inflammasome family could be recruited to form the same platform in bone marrow macrophages infected with Salmonella^48^ or in glomerular infections^49^. Indeed, it has been demonstrated that the activation of inflammasome can occur through dual activation of the NLRP3 and NLR family of caspase recruitment domain-(CARD)-containing protein 4 (NLRC4) platforms. Therefore, it is possible that NLRP3 absence may lead to the NLRC4 increase^48^. The increased cytokine expression in CC from NLRP3^−/−^ mice was associated with an impairment of the erectile function and sodium nitroprusside-induced relaxation in CC. Taken together, these results suggest that compensatory changes induced by the NLRP3 deletion in CC may account for the differences observed in functional responses between NLRP3 pharmacological inhibition and NLRP3^−/−^ mice.

Our next step was to investigate whether the pharmacological inhibition would cause the same effects observed after its genetic inhibition upon the cavernosal functional responses. The small molecule MCC950 is a potent and selective inhibitor of NLRP3. Coll and co-workers^32^ demonstrated that MCC950, at nanomolar concentrations, inhibits NLRP3, but not other inflammasomes, such as AIM2, NLRC4, and NLRP1. Furthermore, MCC950 reduced IL-1β production *in vivo* and rescued the neonatal lethality in a mouse model of the cryopyrin-associated periodic syndrome, and it was effective in *ex vivo* samples from individuals with Muckle-Wells syndrome^32^. Both syndromes are characterized by four different missense mutations in the exon 3 of the NLRP3 gene, which cause gain-of-function and defines NLRP3 as a critical component of the inflammatory process^50^.

Following the previous idea, in this study, the pharmacological inhibition of NLRP3 did not change basal caspase-1 activation and IL-1β release. This result could indicate that NLRP3 is not the sole responsible for the maintenance of the basal levels of caspase-1 and IL-1β. Also, it suggests that another member of the inflammasome family may partially assume NLRP3 function after its inhibition. NLRP3 activation is mainly driven by oxidative stress^51^ and cytokines release^52^. Also, its activation is closely linked to vascular function impairment^53^ and to the generation and/or worsening of cardiovascular^54^ and metabolic^55^ diseases, such as arterial hypertension^56,57^, atherosclerosis^58^, diabetes^59^, and obesity^60,61^. These effects occur because increased IL-1β or IL-18 cytokines promote endothelial dysfunction^62,63^ and vascular smooth muscle proliferation^64,65^. In contrast, the present study demonstrated that NLRP3 inhibition impaired the endothelium-dependent and endothelium-independent relaxation.

The canonical activation of NLRP3 uses the apoptosis-associated speck-like protein containing CARD (ASC), an adaptor protein, to activate caspase-1 and, subsequently, the release of IL-1β and IL-18^66^. On the other hand, the non-canonical activation of NLRP3 is mediated by caspase-11, which triggers caspase-1-independent macrophage death and caspase-1-dependent IL-1β and IL-18 production in response to inflammasome activators. Caspase-11 is expressed not only in cells of the immune system but also in the epithelium^67–69^. The present study indicates that NLRP3 may modulate the cavernosal smooth muscle relaxation, at least partially, independent of its canonical and noncanonical role, since MCC950 did not inhibit caspase-1 and IL-1β production at basal conditions.

The NO is synthesized by the constitutive forms of NOS: the nNOS and eNOS. These enzymes are coupled to Ca^2+^ and calmodulin and are involved in the relaxation of CC. NO-induced soluble guanylyl cyclase (GC) stimulation is essential in the erectile process, and it has been reviewed in detail^70,71^. GC catalyzes the conversion of guanosine triphosphate (GTP) into cyclic guanosine monophosphate (cGMP). cGMP activates the PKG1, promotes depletion of cytosolic calcium (Ca^2+^), and this leads to CC smooth muscle relaxation^6,72,73^. NO may be also produced by the inducible NOS (iNOS) isoform, which is expressed in inflammatory condition such as endotoxemia induced by LPS treatment^74^.

Surprisingly NLRP3^−/−^ mice displayed increased eNOS phosphorylation and GCβ protein expression in CC. Conversely, the pharmacological inhibition of NLRP3 with MCC950 impaired CC relaxation. In conjunction, there was a reduction in GCβ subunit expression, which may account for cavernosal decreased relaxation. NO/cGMP pathway has an anti-inflammatory effect by reducing the expression of intracellular cell adhesion molecule-1 (ICAM-1) and vascular cell adhesion molecule-1 (VCAM-1) induced by TNF-α in rat aorta^75^ or in carrageenan model of hypernociception^76^. Also, NO inhibits the NLRP3 inflammasome activation in macrophages, which may involve S-nitrosylation of NLRP3 and caspase-1^77^. Therefore, it is tempting to speculate that the increased eNOS phosphorylation and GCβ may occur to counteract the increased expression of IL-1β in the CC of NLRP3^−/−^ mice. On the other hand, the PKG1 expression is reduced in the CC of NLRP3^−/−^ mice, and this could indicate that NLRP3 modulates the NO-dependent relaxation in CC.

Experiments were also performed to determine whether the NLRP3 activation would promote opposite effects from those after NLRP3 genetic deletion or its pharmacological inhibition. Indeed, CC stimulation with LPS + ATP (NLRP3 activation) decreased ACh-(endothelium-dependent) and SNP-(endothelium-independent)-induced relaxation. High cytokine levels lead to increased ROS generation and impair the NO/cGMP pathway^62,78^. Based on these results we speculate that increased caspase-1 and IL-1β may lead to endothelial and smooth muscle dysfunction, which then underlie cavernosal reactivity dysfunction. Further support to this idea is the fact that CC from NLRP3^−/−^ mice, which exhibited increased caspase-1 and IL-1β, also displayed reduced relaxation to a NO donor. However, it is noteworthy that the NLRP3 activation was performed with ATP (as a second signal to activate NLRP3) and mice CC not only express purinergic receptors but also respond to their activation. ATP decreases phenylephrine-induced contraction in preparations of CC from rabbits^79,80^ and acts as a potent relaxant agent in CC from humans^81^. Also, the sequential hydrolysis of ATP may result in adenosine formation, which directly relaxes mice CC^82,83^. Therefore, ATP and other metabolic breakdown products may account for some of the effects observed in the present study. The development of pharmacological tools (agonists) more specific to activate NLRP3 in CC will enable to rule out this possibility. On the other hand, LPS + ATP increased the relaxation response to EFS. The relaxation produced by EFS is mainly dependent on nNOS activity. Previous studies have shown inconsistent results on nNOS expression after LPS stimulation. As an example, the expression of nNOS increased in rat oligodendrocytes^84^ and paraventricular nucleus^85^, and vena cava^86^ of pigs. Nevertheless, nNOS expression decreased in rat cardiac myocytes after LPS^87^ incubation. In the present study, LPS + ATP stimulation did not change nNOS expression in mice CC, suggesting that nNOS is not involved in the increased functional response to EFS-induced relaxation. Therefore, additional studies are required to investigate the mechanisms responsible for increased CC relaxation evoked by EFS after LPS + ATP stimuli.

NLRP3 activation reduced the activity of eNOS and expression of GCβ in CC. Indeed, increased activity of NLRP3 impairs the endothelial function in the vasculature through aldosterone-^31^ or TXNIP-induced NLRP3 activation^88,89^. Also, NLRP3 increased activity in the endothelium synergizes with hyperlipidemia to cause a topographic distribution of atherosclerotic lesions^90^. Considering that GCβ is stimulated by NO, the reduction of its expression might be due to the decrease in eNOS activity.

In summary, our study shows that NLRP3 has a dual role in mice CC relaxation *in vitro*, with its inhibition leading to impairment of nitric oxide-mediated relaxation, while its activation by LPS + ATP causes decreased cavernosal smooth muscle sensitivity to NO and endothelium-dependent relaxation. Therefore, NLRP3 may represent a novel target to modulate erectile function.

## Supplementary information

Supplementary Data
