# Author Correction: The inflammasome NLRP3 plays a dual role on mouse corpora cavernosa relaxation

**DOI:** 10.1038/s41598-020-58146-9

**Published:** 2020-01-21

**Authors:** Rafael S. Fais, Fernanda L. Rodrigues, Camila A. Pereira, Allan C. Mendes, Fabíola Mestriner, Rita C. Tostes, Fernando S. Carneiro

**Affiliations:** 1 Departments of Pharmacology, Ribeirao Preto Medical School, University of Sao Paulo, Sao Paulo, Brazil; 2 Departments of Physiology, Ribeirao Preto Medical School, University of Sao Paulo, Sao Paulo, Brazil

Correction to: *Scientific Reports* 10.1038/s41598-019-52831-0, published online 07 November 2019

The Acknowledgements section in this Article was omitted. The Acknowledgements section should read:

“This work was supported by the Sao Paulo Research Foundation (FAPESP, 2016/11988-5; 2016/07641-0; 2018/05638-7)”.

