# Supplementary material for: The inflammasome NLRP3 plays a dual role on mouse corpora cavernosa relaxation
Source: Sci Rep. 2019 Nov 7;9:16224. doi: 10.1038/s41598-019-52831-0 (PMC6838322; doi:10.1038/s41598-019-52831-0)
Supplement: Supplementary file 1 — Supplementary Data [file 41598_2019_52831_MOESM1_ESM.pdf]

The inflammasome NLRP3 plays a dual role on mouse corpora cavernosa relaxation

Rafael S Fais[1], Fernanda L Rodrigues[2], Camila A Pereira[1], Allan C Mendes[1], Fabíola Mestriner[1], Rita C Tostes[1], Fernando S Carneiro[1].

Departments of [1] Pharmacology and [2] Physiology, Ribeirao Preto Medical School, University of Sao Paulo.

Short title:

Corresponding Author: Fernando Silva Carneiro, Ph.D., Department of Pharmacology, Ribeirao Preto Medical School – University of Sao Paulo, Av. Bandeirantes, 3900, 14049-900, Ribeirao Preto, SP, Brazil. Phone: +55(16)33153046; Fax: +55(16)33150220; fsilvac@usp.br

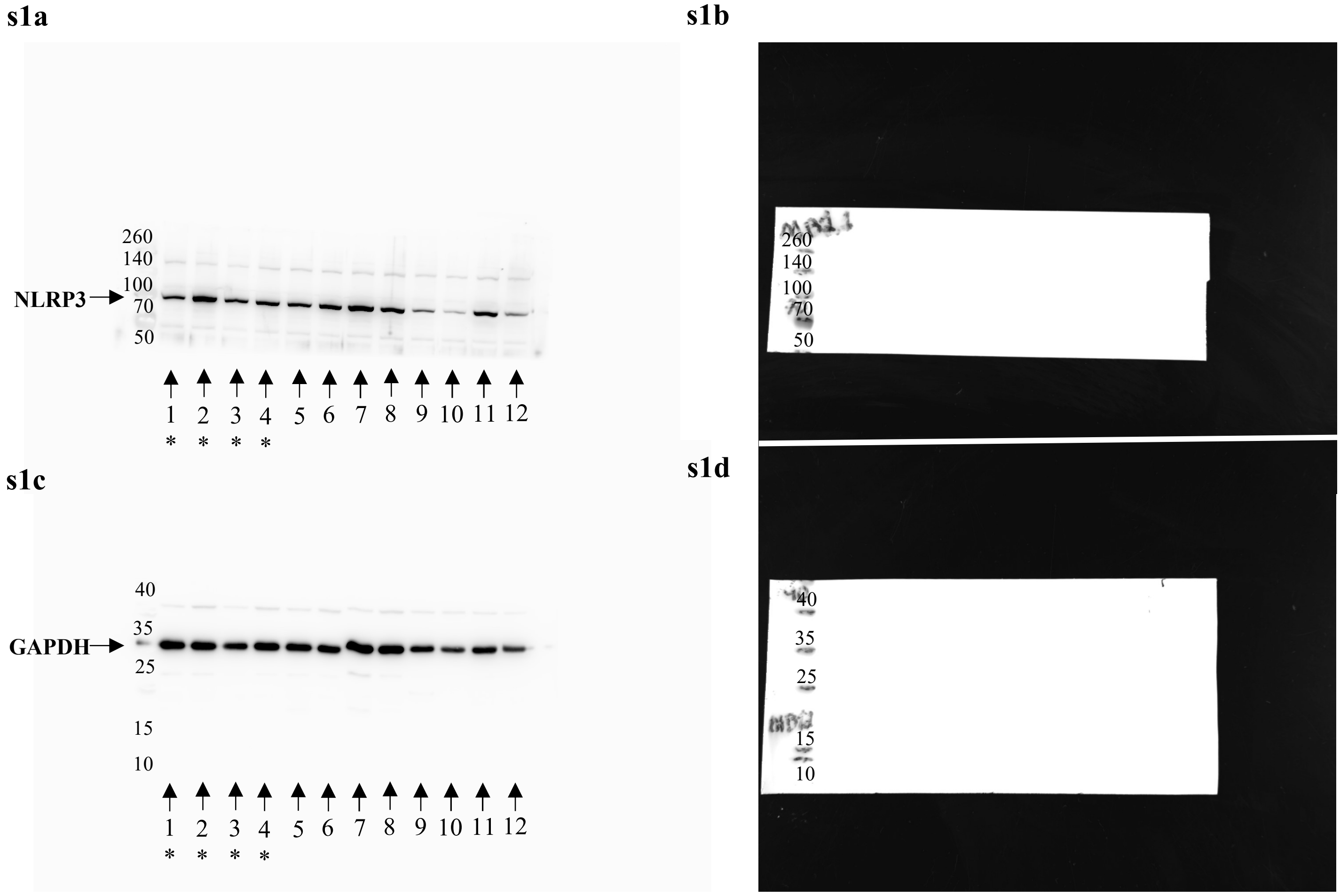

Lane 1: WT CC vehicle 4h
Lane 2: WT CC LPS+ATP
Lane 3: WT CC vehicle 2h
Lane 4: WT CC MCC950

Lane 5: WT CC vehicle 4h
Lane 6: WT CC LPS+ATP
Lane 7: WT CC vehicle 2h
Lane 8: WT CC MCC950

Lane 9: NLRP3$^{-/-}$ CC
Lane 10: WT CC
Lane 11 : NLRP3$^{-/-}$ CC
Lane 12: WT CC

Figure s1. Original membrane showing the NLRP3 bands obtained by chemiluminescence **(a)**, the same membrane visualized by epi-luminescence **(b)**. Original membrane showing the GAPDH bands obtained by chemiluminescence **(c)**, the same membrane visualized by epi-luminescence **(d)**. * Represents the bands used for the representative images.

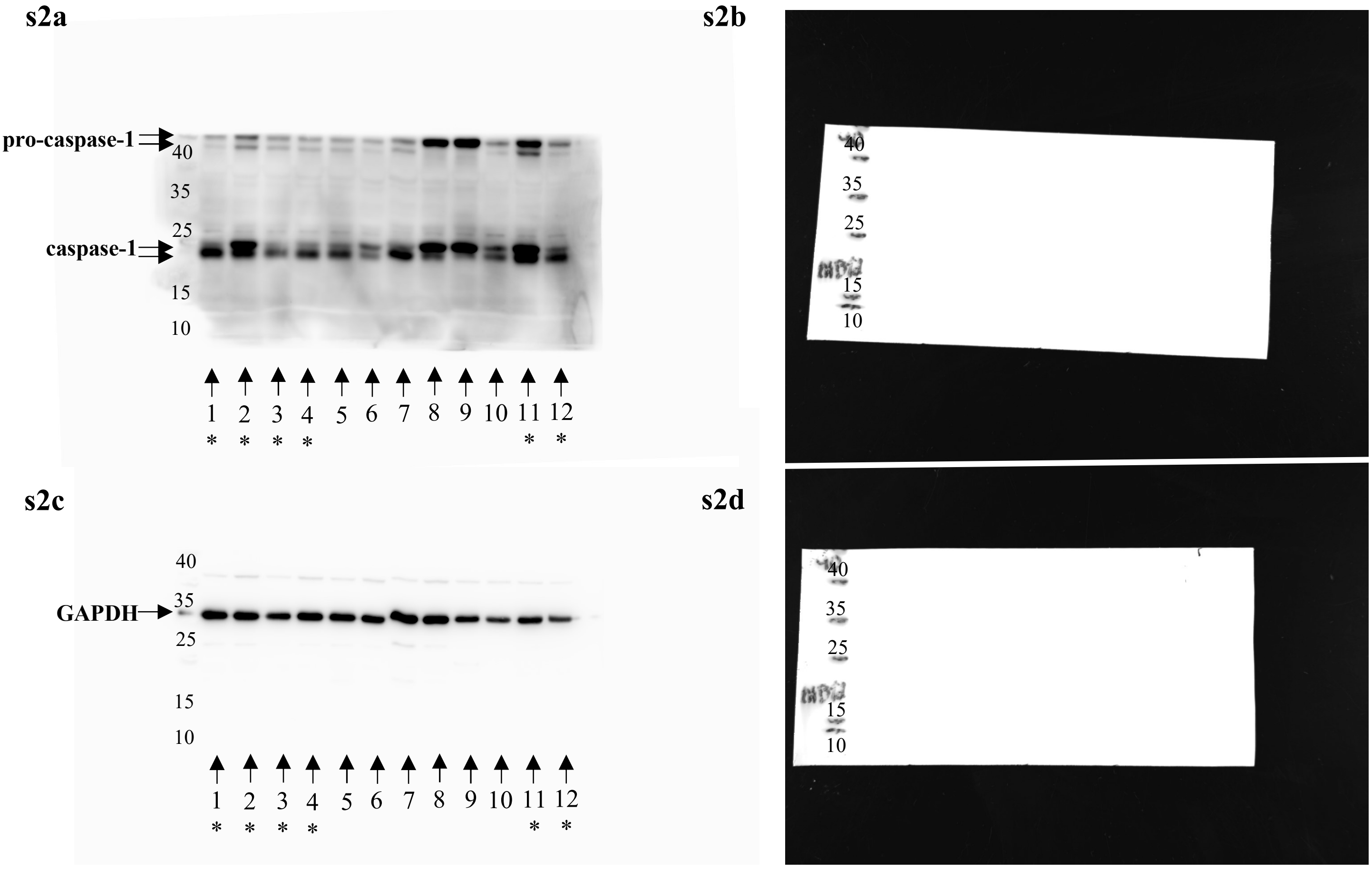

Lane 1: WT CC vehicle 4h
Lane 2: WT CC LPS+ATP
Lane 3: WT CC vehicle 2h
Lane 4: WT CC MCC950

Lane 5: WT CC vehicle 4h
Lane 6: WT CC LPS+ATP
Lane 7: WT CC vehicle 2h
Lane 8: WT CC MCC950

Lane 9: NLRP3$^{-/-}$ CC
Lane 10: WT CC
Lane 11 : NLRP3$^{-/-}$ CC
Lane 12: WT CC

Figure s2. Original membrane showing the pro-caspase-1 and caspase-1 bands obtained by chemiluminescence **(a)**, the same membrane visualized by epi-luminescence **(b)**. Original membrane showing the GAPDH bands obtained by chemiluminescence **(c)**, the same membrane visualized by epi-luminescence **(d)**. * Represents the bands used for the representative images.

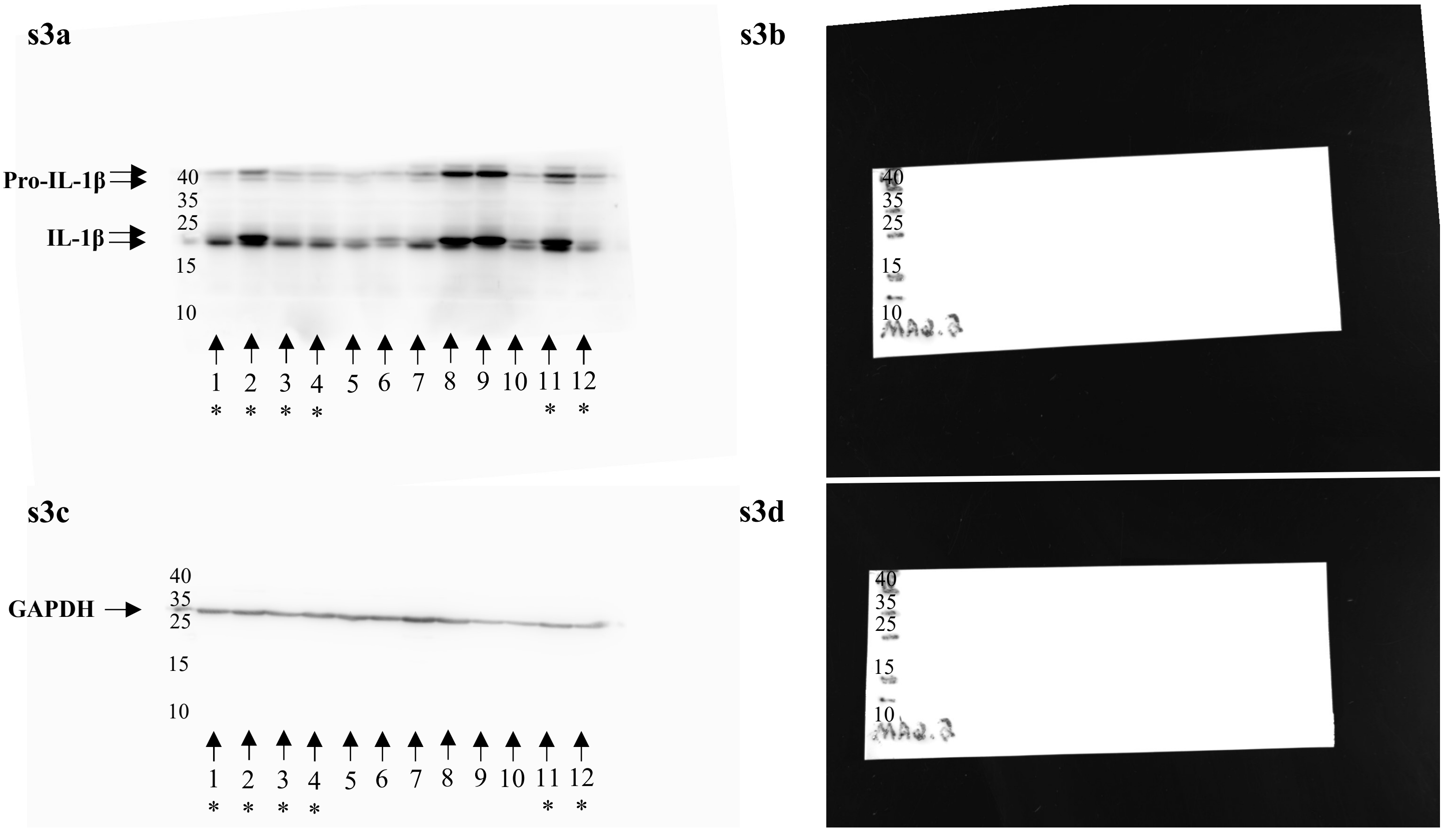

Lane 1: WT CC vehicle 4h
Lane 2: WT CC LPS+ATP
Lane 3: WT CC vehicle 2h
Lane 4: WT CC MCC950

Lane 5: WT CC vehicle 4h
Lane 6: WT CC LPS+ATP
Lane 7: WT CC vehicle 2h
Lane 8: WT CC MCC950

Lane 9: NLRP3$^{-/-}$ CC
Lane 10: WT CC
Lane 11 : NLRP3$^{-/-}$ CC
Lane 12: WT CC

Figure s3. Original membrane showing the pro-IL-1β and IL-1β bands obtained by chemiluminescence **(a)**, the same membrane visualized by epi-luminescence **(b)**. Original membrane showing the GAPDH bands obtained by chemiluminescence **(c)**, the same membrane visualized by epi-luminescence **(d)**. * Represents the bands used for the representative images.

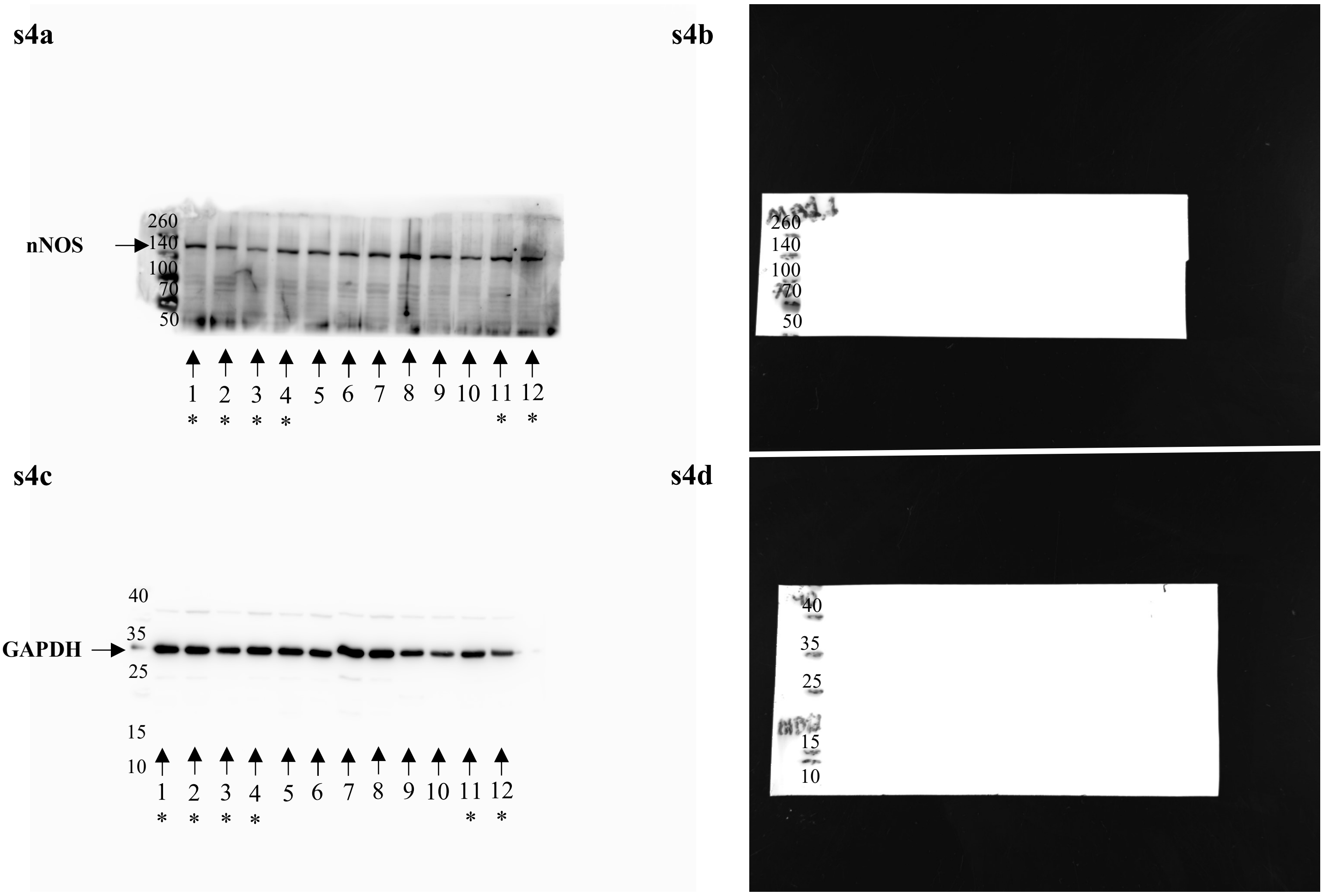

Lane 1: WT CC vehicle 4h
Lane 2: WT CC LPS+ATP
Lane 3: WT CC vehicle 2h
Lane 4: WT CC MCC950

Lane 5: WT CC vehicle 4h
Lane 6: WT CC LPS+ATP
Lane 7: WT CC vehicle 2h
Lane 8: WT CC MCC950

Lane 9: NLRP3$^{-/-}$ CC
Lane 10: WT CC
Lane 11 : NLRP3$^{-/-}$ CC
Lane 12: WT CC

Figure s4. Original membrane showing the nNOS bands obtained by chemiluminescence **(a)**, the same membrane visualized by epi-luminescence **(b)**. Original membrane showing the GAPDH bands obtained by chemiluminescence **(c)**, the same membrane visualized by epi-luminescence **(d)**. * Represents the bands used for the representative images.

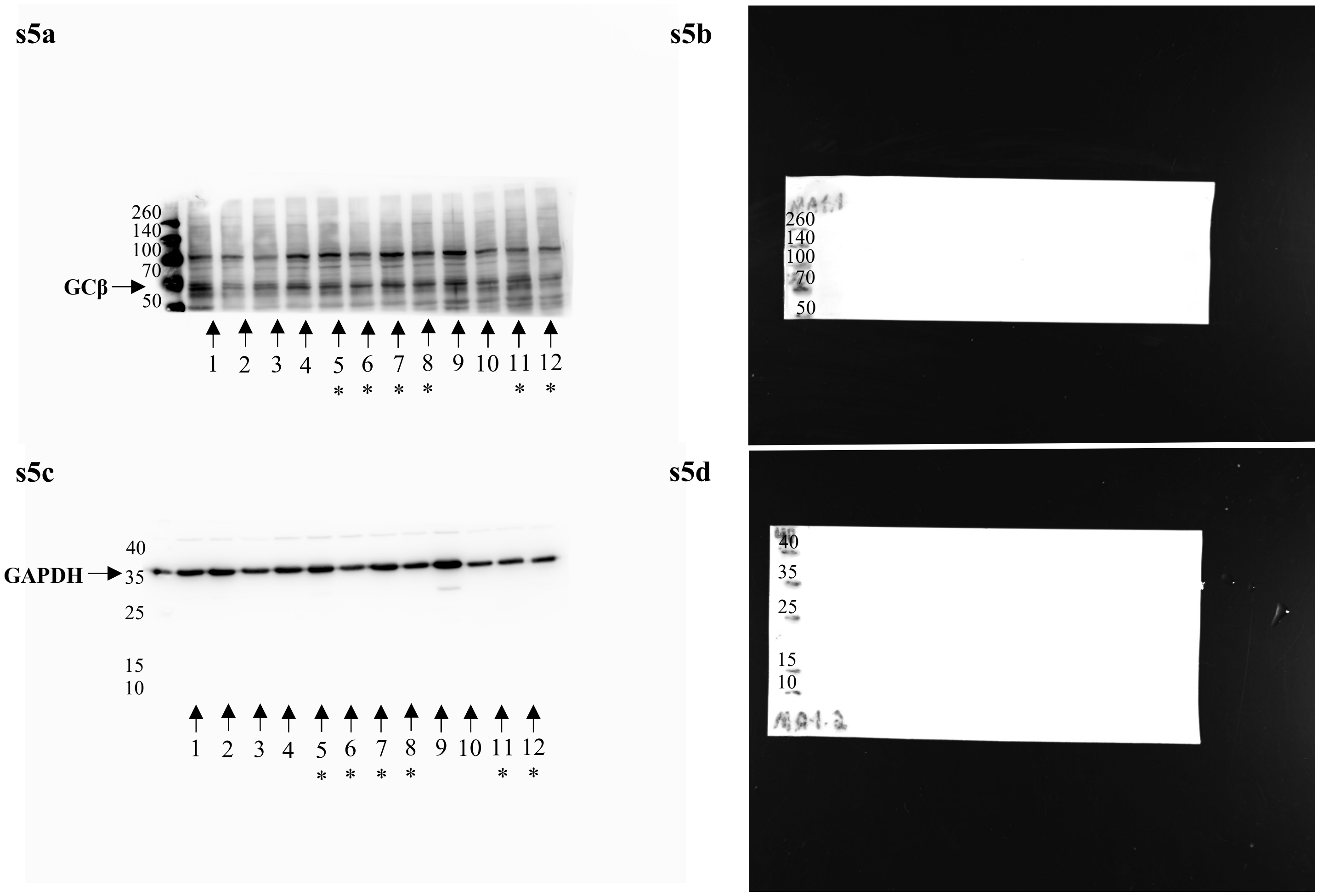

Lane 1: WT CC vehicle 4h
Lane 2: WT CC LPS+ATP
Lane 3: WT CC vehicle 2h
Lane 4: WT CC MCC950

Lane 5: WT CC vehicle 4h
Lane 6: WT CC LPS+ATP
Lane 7: WT CC vehicle 2h
Lane 8: WT CC MCC950

Lane 9: NLRP3$^{-/-}$ CC
Lane 10: WT CC
Lane 11 : NLRP3$^{-/-}$ CC
Lane 12: WT CC

Figure s5. Original membrane showing the GCβ bands obtained by chemiluminescence **(a)**, the same membrane visualized by epi-luminescence **(b)**. Original membrane showing the GAPDH bands obtained by chemiluminescence **(c)**, the same membrane visualized by epi-luminescence **(d)**. * Represents the bands used for the representative images.

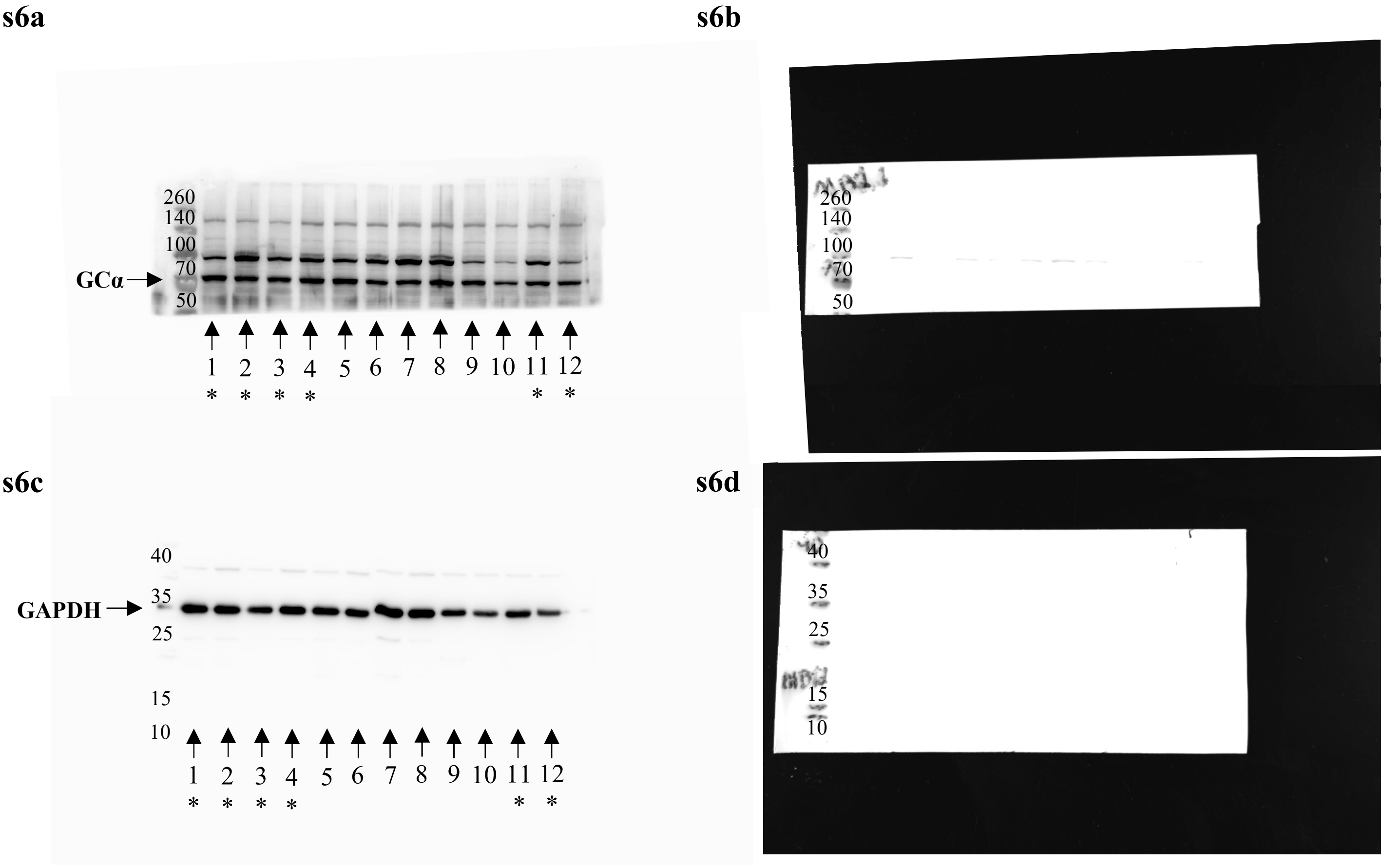

Lane 1: WT CC vehicle 4h
Lane 2: WT CC LPS+ATP
Lane 3: WT CC vehicle 2h
Lane 4: WT CC MCC950

Lane 5: WT CC vehicle 4h
Lane 6: WT CC LPS+ATP
Lane 7: WT CC vehicle 2h
Lane 8: WT CC MCC950

Lane 9: NLRP3$^{-/-}$ CC
Lane 10: WT CC
Lane 11 : NLRP3$^{-/-}$ CC
Lane 12: WT CC

Figure s6. Original membrane showing the GCα bands obtained by chemiluminescence **(a)**, the same membrane visualized by epi-luminescence **(b)**. Original membrane showing the GAPDH bands obtained by chemiluminescence **(c)**, the same membrane visualized by epi-luminescence **(d)**. * Represents the bands used for the representative images.

s7a

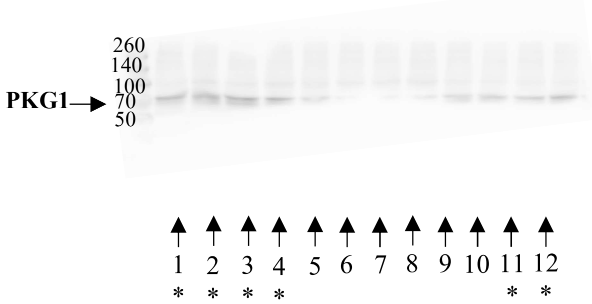

s7b

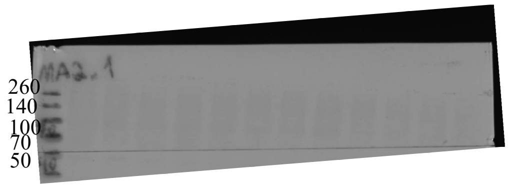

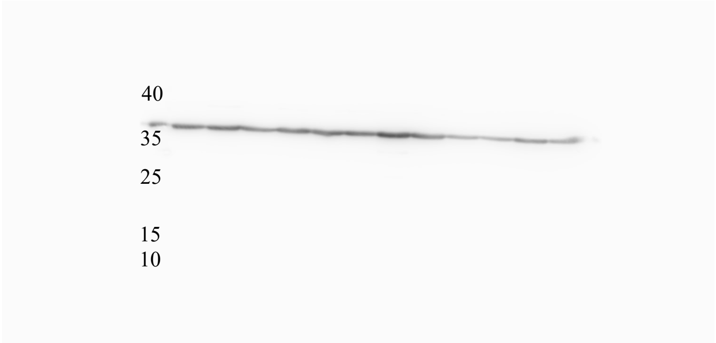

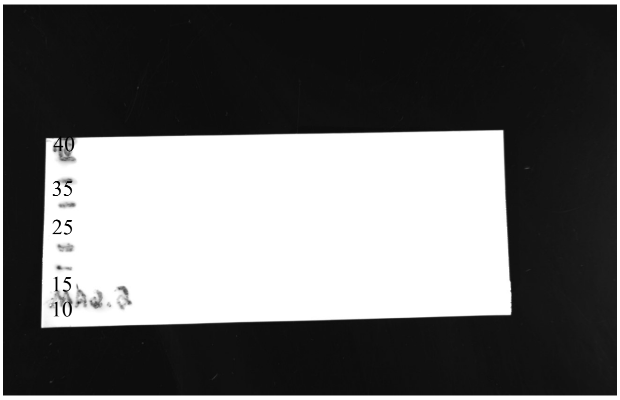

Lane 1: WT CC vehicle 4h
Lane 2: WT CC LPS+ATP
Lane 3: WT CC vehicle 2h
Lane 4: WT CC MCC950

Lane 5: WT CC vehicle 4h
Lane 6: WT CC LPS+ATP
Lane 7: WT CC vehicle 2h
Lane 8: WT CC MCC950

Lane 9: NLRP3$^{-/-}$ CC
Lane 10: WT CC
Lane 11 : NLRP3$^{-/-}$ CC
Lane 12: WT CC

Figure s7. Original membrane showing the PKG1 bands obtained by chemiluminescence **(a)**, the same membrane visualized by epi-luminescence **(b)**. Original membrane showing the GAPDH bands obtained by chemiluminescence **(c)**, the same membrane visualized by epi-luminescence **(d)**. * Represents the bands used for the representative images.

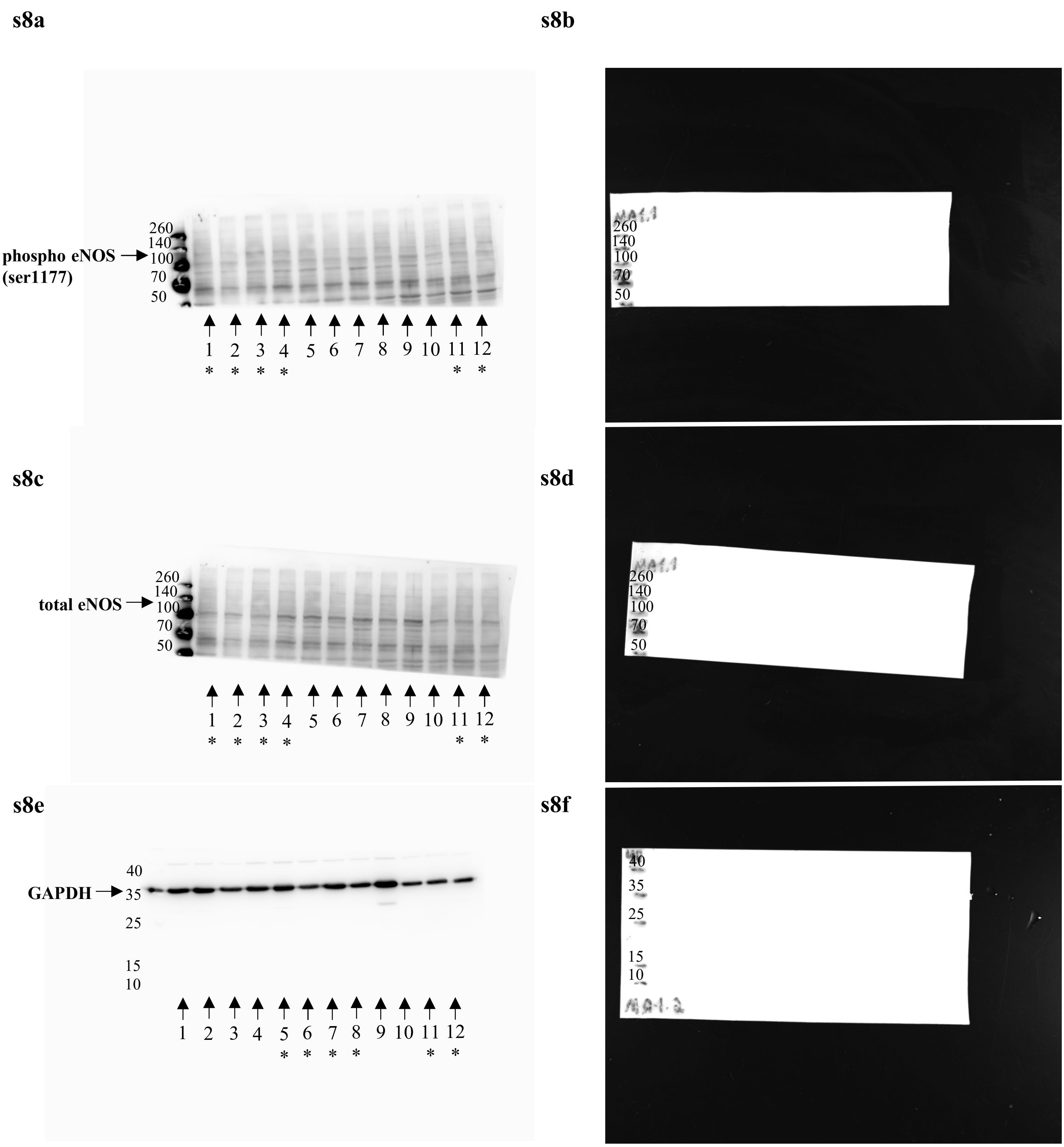

Lane 1: WT CC vehicle 4h
Lane 2: WT CC LPS+ATP
Lane 3: WT CC vehicle 2h
Lane 4: WT CC MCC950

Lane 5: WT CC vehicle 4h
Lane 6: WT CC LPS+ATP
Lane 7: WT CC vehicle 2h
Lane 8: WT CC MCC950

Lane 9: $NLRP3^{-/-}$ CC
Lane 10: WT CC
Lane 11: $NLRP3^{-/-}$ CC
Lane 12: WT CC

Figure s8. Original membrane showing the phospho eNOS (ser1177) bands obtained by chemiluminescence **(a)**, the same membrane visualized by epi-luminescence **(b)**. Original membrane showing the total eNOS bands obtained by chemiluminescence **(c)**, the same membrane visualized by epi-luminescence **(d)** Original membrane showing the GAPDH bands obtained by chemiluminescence **(e)**, the same membrane visualized by epi-luminescence **(f)**. * Represents the bands used for the representative images.

s9a

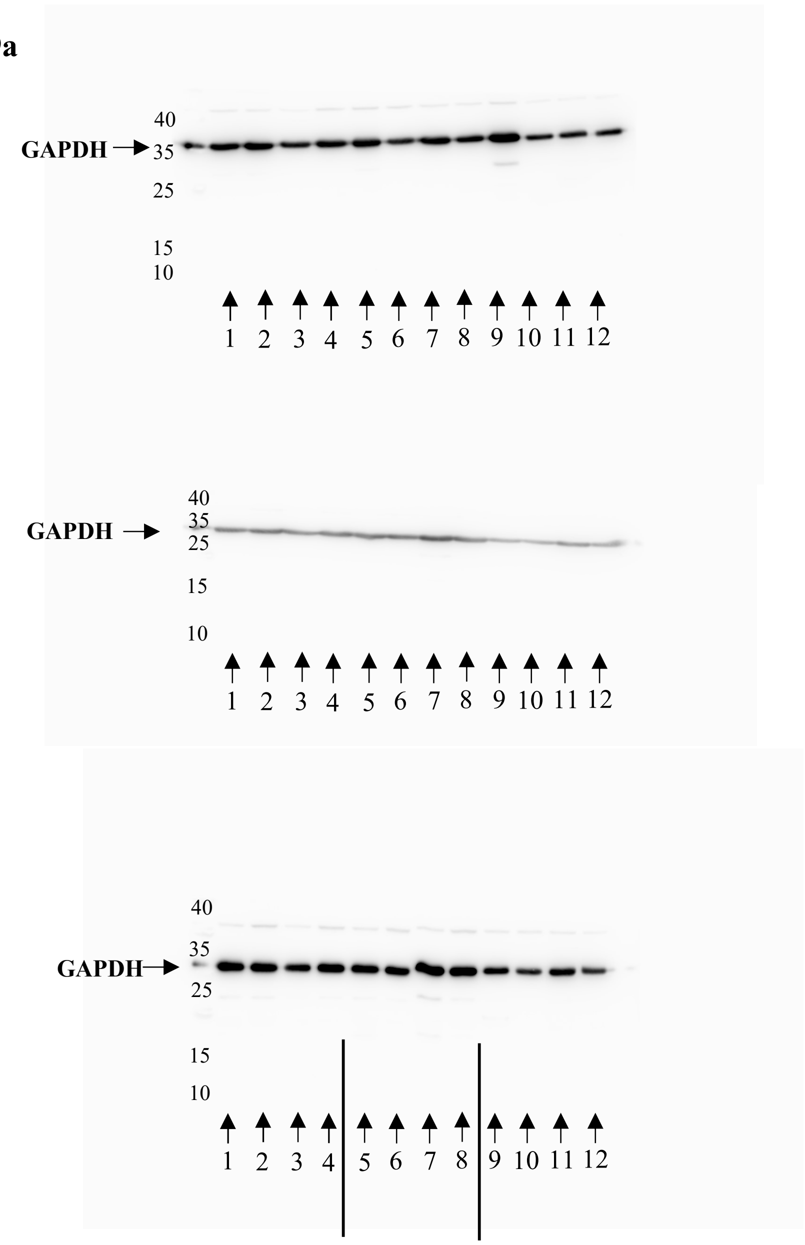

Lane 1: WT CC vehicle 4h
Lane 2: WT CC LPS+ATP
Lane 3: WT CC vehicle 2h
Lane 4: WT CC MCC950
Lane 5: WT CC vehicle 4h
Lane 6: WT CC LPS+ATP
Lane 7: WT CC vehicle 2h
Lane 8: WT CC MCC950
Lane 9: NLRP3$^{-/-}$ CC
Lane 10: WT CC
Lane 11: NLRP3$^{-/-}$ CC
Lane 12: WT CC

s9b

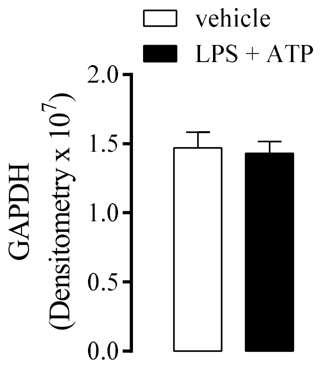

s9c

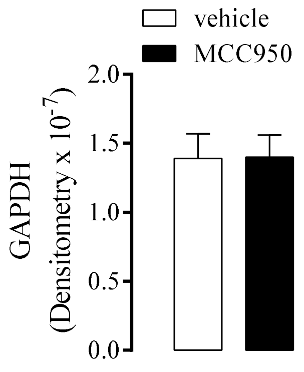

s9d

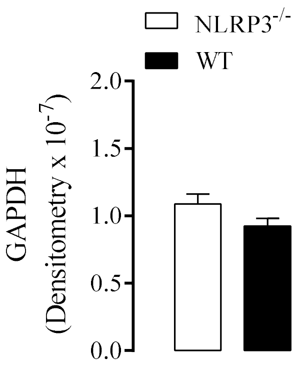

Figure s9. Original membranes showing the GAPDH bands obtained by chemiluminescence (**a**) Densitometric analysis of GAPDH RAW data expression in CC strips of WT mice incubated with LPS + ATP (1 µg/mL +2 nM, black bar) or vehicle (white) bar for 4 hours (**b**); MCC950 (1µM, black bar) or vehicle (white bar) for 2 hour (**c**); CC strips od WT (white bar) or NLRP3$^{-/-}$ (black bar) (**d**) mice. The bars represent the mean ± SEM values of protein expression. *, # $p<0.05$ compared to respectively vehicle incubation 2 hours and 4 hours group. n=6. The comparison of protein expression was performed by Student's t-test.

s10a

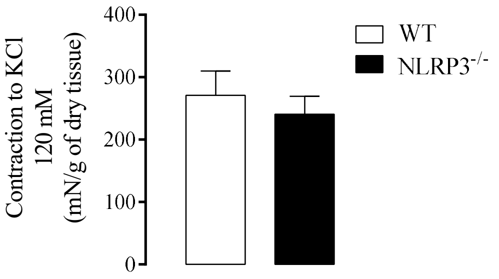

s10b

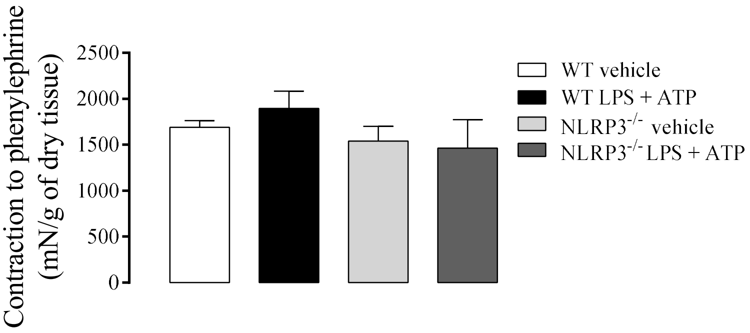

s10c

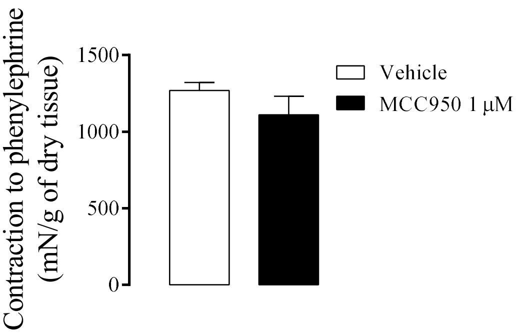

Figure s10. Contraction to KCl 120 mM (a), pre-contraction to phenylephrine (10μM) after the incubation with LPS + ATP (1 μg/mL + 2 nM) for 4 hours (b) or MCC950 (1μM) for 2 hours (c) in CC strips of WT and NLRP3$^{-/-}$ mice. The bars represent the mean ± SEM values of protein expression. n=6. The comparison of protein expression was performed by Student's t-test (a and c) or ANOVA followed by Tukey (b).
